# A *piggyBac*-based platform for genome editing and clonal rhesus macaque iPSC line derivation

**DOI:** 10.1038/s41598-021-94419-7

**Published:** 2021-07-29

**Authors:** Ignacio Rodriguez-Polo, Sophie Mißbach, Stoyan Petkov, Felix Mattern, Anna Maierhofer, Iga Grządzielewska, Yuliia Tereshchenko, Daniel Urrutia-Cabrera, Thomas Haaf, Ralf Dressel, Iris Bartels, Rüdiger Behr

**Affiliations:** 1 Research Platform Degenerative Diseases, German Primate Center-Leibniz Institute for Primate Research, Kellnerweg 4, 37077 Göttingen, Germany; 2 German Center for Cardiovascular Research (DZHK), Partner site Göttingen, Göttingen, Germany; 3 Institut für Humangenetik, Universität Würzburg, Biozentrum, Am Hubland, 97074 Würzburg, Germany; 4 Max Planck Molecular Biology Program (M.Sc./Ph.D.), Justus-von-Liebig-Weg 11, 37077 Göttingen, Germany; 5 Institute of Cellular and Molecular Immunology, University Medical Center Göttingen, Humboldtalle 34, 37073 Göttingen, Germany; 6 Institute of Human Genetics, University Medical Center Göttingen, Robert-Koch-Str. 40, 37075 Göttingen, Germany; 7 Present Address: Cellular Reprogramming Unit, Center for Eye Research Australia, 75 Commercial Road, Melbourne, 3004 Australia

**Keywords:** Biotechnology, Stem-cell biotechnology

## Abstract

Non-human primates (NHPs) are, due to their close phylogenetic relationship to humans, excellent animal models to study clinically relevant mutations. However, the toolbox for the genetic modification of NHPs is less developed than those for other species like mice. Therefore, it is necessary to further develop and refine genome editing approaches in NHPs. NHP pluripotent stem cells (PSCs) share key molecular signatures with the early embryo, which is an important target for genomic modification. Therefore, PSCs are a valuable test system for the validation of embryonic genome editing approaches. In the present study, we made use of the versatility of the *piggyBac* transposon system for different purposes in the context of NHP stem cell technology and genome editing. These include (1) Robust reprogramming of rhesus macaque fibroblasts to induced pluripotent stem cells (iPSCs); (2) Culture of the iPSCs under feeder-free conditions even after removal of the transgene resulting in transgene-free iPSCs; (3) Development of a CRISPR/Cas-based work-flow to edit the genome of rhesus macaque PSCs with high efficiency; (4) Establishment of a novel protocol for the derivation of gene-edited monoclonal NHP-iPSC lines. These findings facilitate efficient testing of genome editing approaches in NHP-PSC before their in vivo application.

## Introduction

Cardiovascular and neurodegenerative diseases are the primary causes of death worldwide^1,2^. Even though these disorders have a complex etiology, they are often of genetic origin^3,4^. To further understand the development and progression of these diseases, highly predictive model systems need to be established. Besides cell and organoid models, it is necessary to use animal models that share the complexity of the human nervous, immune, and cardiovascular systems^5,6^. Non-human primates (NHPs) as our evolutionary closest relatives are excellent animal models, as reflected by high genetic, physiological, developmental, and metabolic similarity with humans^6–12^.

In comparison with other model organisms, NHP models are less established^10,13^, but the demand for them in biomedicine is strongly increasing^10^. Due to the difficulties and the complexity associated with genetic modifications of NHPs, most studies modelling human diseases use drug- or surgically-induced approaches^5,13–15^. However, in the last years, the emergence of new genome editing-tools like CRISPR–Cas9 has revolutionized the generation of genetically modified animals^16^. The first NHP species that were genetically modified, at that time by classical lentiviral transgenesis, were the marmoset and the rhesus macaque^9,15,17–21^. However, the generation of genetically modified NHPs entails difficulties not encountered in other model species. For instance, NHPs have small litter sizes as well as long gestation and postnatal maturation periods. In consequence, it is challenging and cost-intensive to obtain a high number of embryos^7,13,15^. Furthermore, germline transmission of mutations by chimeric embryo formation upon injection of (genetically modified) NHP-PSCs into primate embryos was not successful so far, in contrast to the mouse. Recent advances in this direction reflect the remaining challenges of the process, that need to be addressed before the successful application of these technologies^22,23^. Many efforts have been made to overcome the biological and technical difficulties by refining and adapting protocols developed for mouse, rat, or bovine to NHPs^9^. Finally, the generation of transgenic NHPs is also ethically controversial^10,24^. All these obstacles make the careful evaluation of genome editing approaches necessary before their application in vivo^25^*.* In order to search for an appropriate in vivo genome editing approach, certain aspects must be critically assessed including its (1) efficacy and efficiency, (2) specificity, (3) translatability, and (4) safety for the NHPs during the experiment. Species-specific pluripotent stem cells are a valuable tool for the in vitro validation at least of the efficiency and specificity of the respective editing approaches.

NHP-PSCs share many features with the pluripotent cells of the early embryo^6,26^. Therefore, they are the best available test system to study embryonic genome editing ex vivo and to predict, upon directed differentiation into specific cell types, potential phenotypic alterations that might be observed later on in the animal. Using this work-flow, it is possible to simultaneously evaluate both, the potential phenotypic consequences for the animals as well as the potential value of the in vivo model.

For DNA-based reprogramming and genome editing, it is crucial to deliver relatively large transgenes with high efficiency. Furthermore, robust long-term expression can be relevant for certain purposes, specifically for reprogramming differentiated cells to iPSCs. Reprogramming requires the exogenous expression of embryonic key transcription factors to awake pluripotency in somatic cells^27,28^. In the case of genome editing, it is necessary to express nucleases plus guide RNAs^28,29^. The *piggyBac* transposon is a mobile genetic element originally identified in a moth (*Trichoplusia ni*) that efficiently transposes between the donor vector and host chromosomes. This system has two major advantages, (1) it has almost no cargo limit, and (2) it is fully reversible, leaving no footprint in the genome after excision^28,30^. The *piggyBac* transposon/transposase system consists of a transposase that recognizes *piggyBac*-specific inverted terminal repeat sequences (ITRs) located on both sides of the transposon cassette. The transposase excises the transposable element to integrate it into TT/AA chromosomal sites. Together, these characteristics make the *piggyBac* an excellent biotechnological tool for testing genetic modifications of NHP^27,30–32^.

Here we show and characterize different applications of the *piggyBac* system, which will facilitate the evaluation of genome editing in vitro before in vivo applications*.* First, the previously published *piggyBac* 6-reprogramming factor construct can efficiently generate iPSCs from adult rhesus macaque fibroblasts^27^. The generated iPSCs can be cultured under feeder-free conditions. We demonstrate that the exogenous expression of the reprogramming construct gets silenced during reprogramming and passaging. To show the stability of the reprogrammed state, we exemplarily removed the transposon from one iPSC line after reprogramming, thereby generating transgene-free macaque iPSCs. Furthermore, we employed the *piggyBac* vector for efficient genome editing in NHP-iPSCs. Finally, we developed a robust protocol for clonal derivation of rhesus macaque PSCs. Combining our tools and protocols, we succeeded in the straightforward establishment of clonal rhesus monkey iPSC lines harboring clinically relevant mutations.

## Results

### Reprogramming adult rhesus macaque skin fibroblasts using the *piggyBac* 6-factors-in-one-vector system

**Figure 1:**
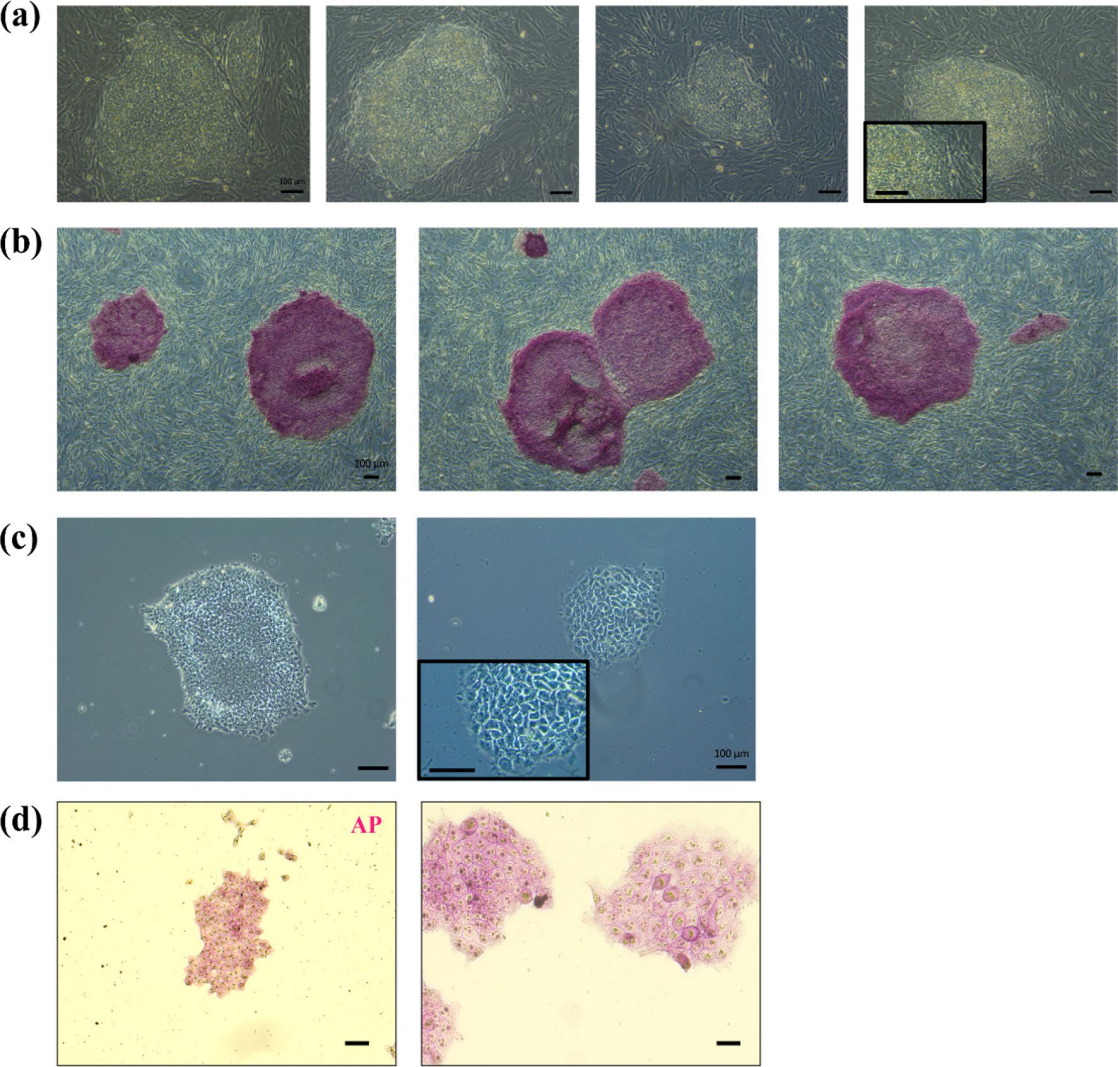
Rhesus induced pluripotent stem cells generated by *piggyBac* transposition. (**a**) Rhesus iPSC morphology on feeder cells (left to right DPZ_iRhpb#1–4), (**b**) Alkaline phosphatase staining (left to right DPZ_iRhpb#1–3), (**c**) PSC adaptation to feeder-free culture conditions. Left image: Rh366.4 ESCs (Rh_ESC), right image: DPZ_iRhpb#4, (**d**) Alkaline phosphatase staining in feeder-free culture. Left image: DPZ_iRhpb#4, right image: Rh_ESC (Scale bars in (**a**–**c**) 100 µm, in (**d**) 50 µm).

Four independent rhesus iPSC lines, named DPZ_iRhpb#1–4, respectively, were generated via *piggyBac* transposition using our previously published 6-factors-in-one-vector transposon system (Fig. 1a)^27^. The reprogramming transposon encodes the marmoset factors SOX2, OCT4A, KLF4, c-MYC, NANOG, and LIN28. Marmoset and rhesus macaque pluripotency factors show a very high degree of conservation on the cDNA and the protein level (Table 1). The iPSC lines were generated from skin fibroblasts from 2 adult macaques (DPZ_iRhpb#1–3, male) (DPZ_iRhpb#4, female). Approximately 20 days after transfection and selection of the fibroblasts, the first colonies emerged; new colonies appeared at least until day 60. Between 100 and 150 colonies were identified per reprogramming experiment (100–150 primary colonies/1 × 10^6^ transfected cells), resulting in a reprogramming efficiency of approximately 0.08–0.12% (~ 16–25 colonies per primary plate; 0.2 × 10^5^ cells per primary plate). Primary colonies showed the typical morphology of human iPSCs in feeder cell culture (not shown). During the first passages, the best colonies were selected by manual picking according to their morphology. Four of the colonies with good morphology were selected in passage 5 to proceed with further passaging and characterization (Fig. 1a). Between passages 10 and 20, the lines became stable, showing almost no differentiation. The first assessment of the pluripotent state of the cells was done by alkaline phosphatase (AP) staining. All generated lines showed AP activity (Fig. 1b).

**Table 1:** Percentage of similarity between marmoset and rhesus macaque reprogramming factors on the cDNA and the protein level. Values are based on for the following sequences: SOX2 (ENSCJAG00000008401; ENSMMUG00000046147), OCT4A (ENSCJAG00000019789; ENSMMUG00000015688), KLF4 (ENSCJAG00000016955; ENSMMUG00000006088), LIN28 (ENSCJAG00000009796; ENSMMUG00000044953), c-MYC (ENSCJAG00000012620; ENSMMUG00000014601), and NANOG (ENSCJAG00000018999; ENSMMUG00000032158).

	cDNA (%)	Protein (%)
SOX2	93.96	99.68
OCT4	85.46	95.63
KLF4	94.56	100.00
LIN28	96.51	99.04
c-MYC	94.65	97.95
NANOG	86.06	94.44

Three iPSC lines, DPZ_iRhpb#2, DPZ_iRhpb#3, DPZ_iRhpb#4, and a rhesus control ESC line (Rh_ESC), were adapted to feeder-free conditions (DPZ_iRhpb#4 and Rh_ESC, Fig. 1c) (Supplementary Fig. 1) using Stem Max iPS-Brew supplemented with 1 µM IWRI and 0.5 µM CHIR. We recently named this formulation universal primate pluripotent stem cell (UPPS) medium^33^. DPZ_iRhpb#4 was adapted at early passage (passage 10) and DPZ_iRhpb#2 and 3 were maintained in feeder conditions for expansion and characterization and adapted to feeder-free conditions at late passages (between passage 50 and 60). Colony morphology was very similar before and after adaptation (Fig. 1c, and Supplementary Fig. 1a,b, compare with Fig. 1a). The colonies of the iPSC and of the ESC lines present a typical compact structure and a high nucleus/cytoplasm ratio. DPZ_iRhpb#4 was cultured for more than 60 passages in feeder-free conditions, DPZ_iRhpb#2 and 3 for more than 10 passages. All cell lines showed undifferentiated morphology and AP activity during adaptation and maintenance in UPPS medium (DPZ_iRhpb#4 and Rh_ESC, Fig. 1d) (DPZ_iRhpb#2 and 3 Supplementary Fig. 1c,d).

**Figure 2:**
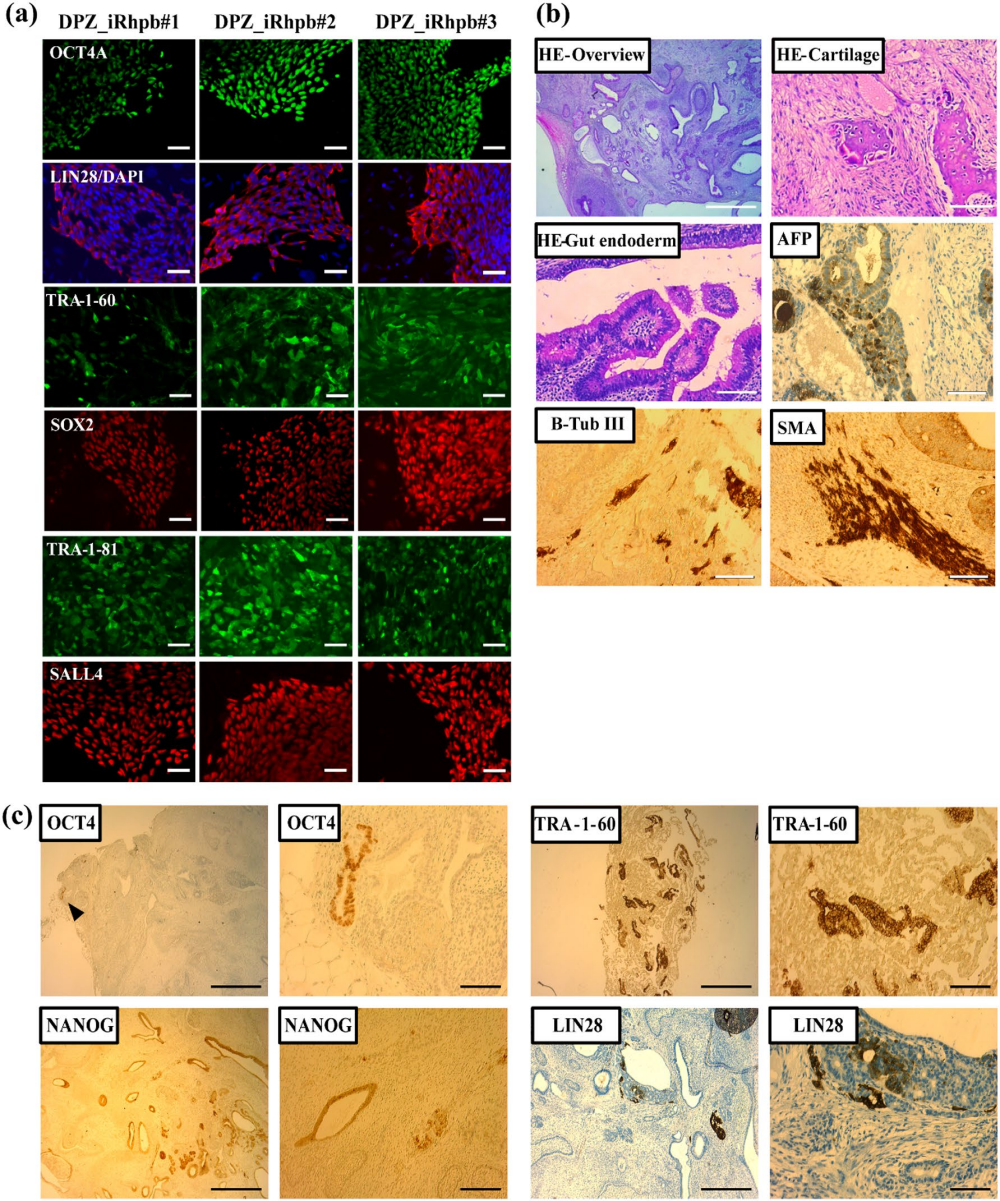
Rhesus iPSC characterization. (**a**) Immunofluorescence staining of Rhesus iPSC colonies of lines DPZ_iRhpb#1–3 in feeder culture. Detection of pluripotency markers OCT4A, LIN28, TRA-1-60, SOX2, TRA-1-81, and SALL4. TRA-1-81, TRA-1-60, and SALL4 are not contained in the reprogramming construct. The origin of OCT4A, LIN28, and SOX2 expression cannot be determined (Scale bar 20 µm). (**b**,**c**) Immunohistochemical analysis of a DPZ_iRhpb#2-derived teratoma. (**b**) Teratoma sections were stained for representative markers of the three germ layers: β-Tubulin III staining indicates ectodermal differentiation. Smooth muscle actin and AFP staining show mesodermal and endodermal differentiation, respectively. HE staining allows the identification of representative tissues. The presence of cartilage indicates mesoderm, and gut endoderm shows endodermal differentiation (Scale bars 100 µm). (**c**) The DPZ_iRhpb#2 teratoma also expressed pluripotency markers OCT4A, NANOG, TRA-1-60, and LIN28. Small isolated clusters were found positive for the pluripotency markers tested (left image of each pair of images: scale bar 1 mm; right image: scale bar 100 µm).

Expression of pluripotency markers was assessed by immunofluorescence staining for all the generated lines. DPZ_iRhpb#1–3 were analyzed in feeder culture (Fig. 2a). Additionally, iPSCs in feeder-free culture were re-analyzed after at least 10 passages in the new culture conditions (DPZ_iRhpb#2, 3, Supplementary Fig. 1e,f) (DPZ_iRhpb#4, Supplementary Fig. 2a). DPZ_iRhpb#1–4 express pluripotency markers OCT4A, LIN28, TRA-1-60, SOX2, TRA-1-81, and SALL4. OCT4A, LIN28, SOX2, and SALL4 were detected in the nucleus, and LIN28 was present in the cytoplasm. TRA-1-60 and TRA-1-81 were present in the membrane. TRA-1-60, TRA-1-81, and SALL4 are pluripotency-related markers not encoded by the transposon, indicating successful reprogramming. Isotype controls were performed as negative control (Supplementary Fig. 2b).

In order to discriminate between the expression of endogenous and exogenous, i.e., *piggyBac*-encoded, pluripotency factors, we performed RT-PCR (Supplementary Fig. 3). Primers were designed to specifically amplify the endogenous transcripts of *OCT4A, SOX2, NANOG*, and *c-MYC*. Primers to evaluate exogenous expression were designed to amplify the fused *LIN28-NANOG* transcript. None of the four lines analyzed showed silencing of the reprogramming construct on the transcript level. Nevertheless, all lines showed reactivation of the endogenous pluripotency genes. The intensity of the PCR bands representing the endogenous transcripts was similar to the respective bands obtained with Rh_ESC (Supplementary Fig. 3).

### In vivo and in vitro assessment of pluripotency

In order to functionally demonstrate pluripotency of DPZ_iRhpb#1–4, teratoma formation assay was performed. All four lines formed teratomas (Fig. 2b, DPZ_iRhpb#2) (Supplementary Fig. 4 DPZ_iRhpb#1 and 3, Supplementary Fig. 5a, DPZ_iRhpb#4). All tumors present a high degree of histological heterogeneity, i.e., differentiation. Cartilage, ossification, smooth muscle cells and gut endothelium were easily histologically identifiable, indicating mesodermal and endodermal differentiation, respectively (Fig. 2b) (Supplementary Fig. 4). Neural tissues positive for β-III tubulin were identified by immunostaining, indicating ectodermal differentiation. In addition, α-smooth muscle actin (SMA) and α-fetoprotein (AFP) stained clusters were present, confirming mesodermal and endodermal differentiation, respectively (Fig. 2b) (Supplementary Figs. 4, 5a). In summary, the teratoma analysis demonstrates pluripotency of the iPSCs.

In order to evaluate if the expression of the pluripotency factors was downregulated during differentiation, teratomas were stained for pluripotency markers. OCT4A, NANOG, TRA-1-60, and LIN28 expression was absent from most cells of the teratomas and was limited to a few isolated clusters found in all teratomas (DPZ_iRhpb#2, Fig. 2c) (DPZ_iRhpb#1, DPZ_iRhpb#3, data not shown). This finding shows that endogenous (OCT4A, NANOG, TRA-1-60, and LIN28) as well as *piggyBac*-dependent (OCT4A, NANOG, and LIN28) pluripotency factor expression is generally downregulated on the protein level during differentiation (Fig. 2c). Isotype controls were performed as negative control (Supplementary Fig. 5b).

Teratoma formation assay was performed with DPZ_iRhpb#1, DPZ_iRhpb#2, DPZ_iRhpb#3 cultured on feeder cells and DPZ_iRhpb#4 in feeder-free conditions. In order to confirm that all iPSC lines maintain their differentiation potential in feeder-free conditions, we performed the embryoid body (EB) formation assay. DPZ_iRhpb#2, DPZ_iRhpb#3, and DPZ_iRhpb#4 were able to differentiate into representative cell types of the three germ layers. Successful differentiation was evaluated by immunostaining for β-III tubulin, SMA and AFP (DPZ_iRhpb#3 and DPZ_iRhpb#4, Supplementary Fig. 5c,d) (DPZ_iRhpb#2 data not shown). Finally, we also evaluated the presence of cells positive for pluripotency markers after in vitro differentiation. All cells were negative for OCT4A, and only two isolated clusters of TRA-1-60 positive cells were found in the culture, confirming our findings in the teratomas (DPZ_iRhpb#3, Supplementary Fig. 5e).

### *piggyBac* silencing during reprogramming and differentiation

**Figure 3:**
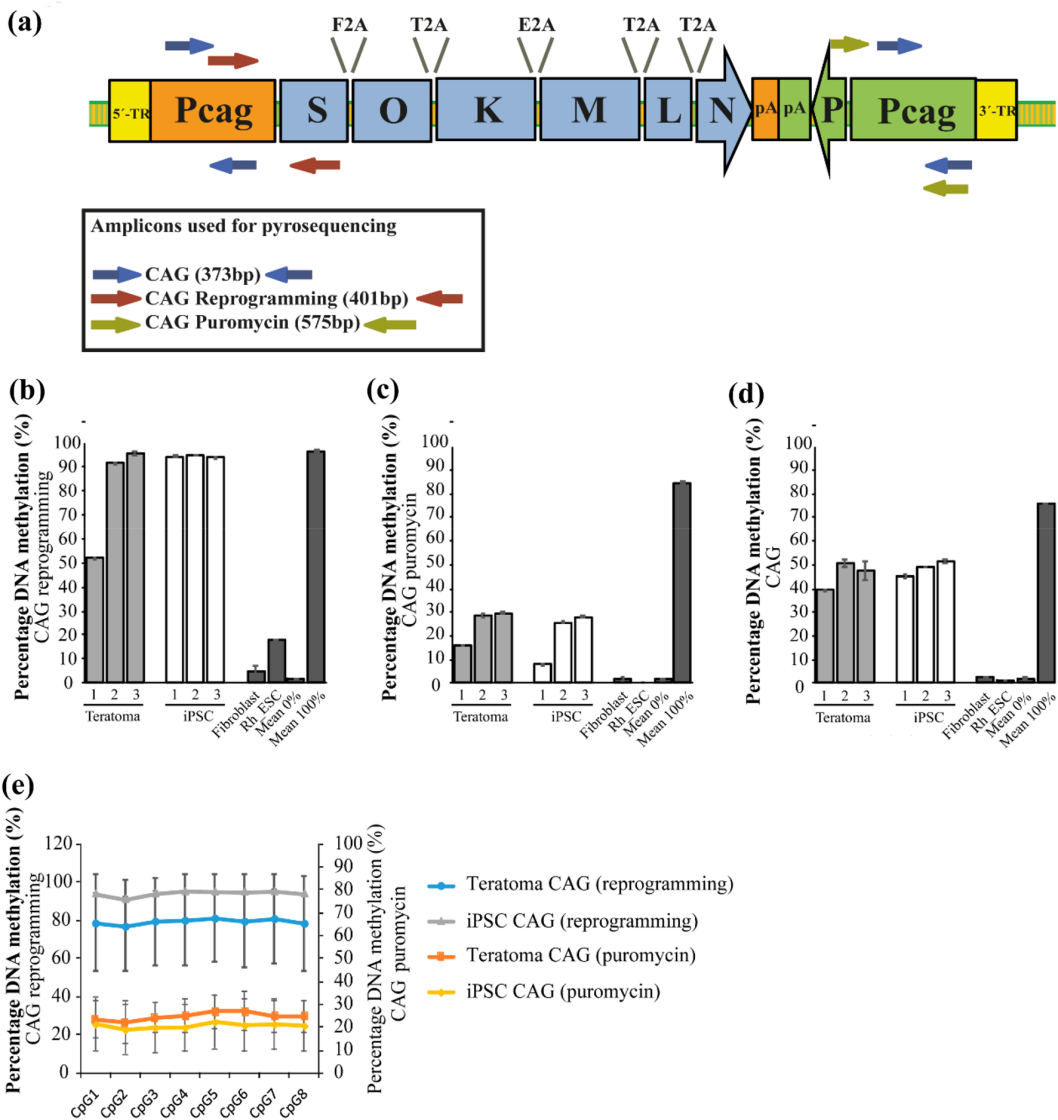
Methylation analysis of the reprogramming construct. (**a**) Schematic of the *piggyBac* reprogramming construct used in this study containing the marmoset pluripotency factors *SOX2* (S), *OCT4* (O), *KLF4* (K), *c-MYC* (M), *LIN28* (L), and *NANOG* (N)^27^. Stop codons were substituted by 2A peptide sequences (F2A, T2A, and E2A). Expression of the reprogramming transcript is driven by a CAG promoter. An independent CAG promoter controls the expression of the puromycin resistance gene (P). Below the vector: Schematic representation of the amplicons used for pyrosequencing. Three different primer combinations were used, one to amplify both CAG promoters (CAG, blue) simultaneously, one to amplify specifically the CAG promoter driving reprogramming cassette expression (CAG reprogramming, red), and one specific for the promoter associated with the puromycin resistance gene (CAG puromycin, green). (**b**–**d**) Methylation analysis of three teratomas (Teratoma 1–3, DPZ_iRhpb#1–3), and three iPSC lines (DPZ_iRhpb#1–3). Two samples were included as negative control: Fibroblasts and Rh_ESCs. Internal technical positive controls are also shown (Mean 0% and Mean 100%) (mean ± SD). (**b**) CAG reprogramming methylation analysis. (**c**) CAG Puromycin methylation analysis. (**d**) Non-discriminative CAG promoter methylation analysis. (**e**) CAG reprogramming vs. CAG puromycin methylation of the 8 CpG sites considered (left y-axis: CAG reprogramming; right y-axis: CAG puromycin). Represented Mean ± SD of the teratomas and the iPSC.

The histological analysis of the teratomas generally suggested silencing of the *piggyBac* transposon used for reprogramming; most of the cells neither expressed OCT4A nor NANOG. For both proteins, we have established very specific and sensitive IHC detection protocols^34^. In order to check if silencing occurs during reprogramming or differentiation, we performed comparative methylation analysis of the reprogramming construct in iPSCs and teratomas (Fig. 3) (Supplementary Fig. 6).

The reprogramming construct contains two separate CAG promoters; one driving the reprogramming factor cassette and one driving the puromycin resistance gene (Fig. 3a). We aimed at getting an overview of the global methylation of the construct in the cultures and gaining insights into possible differential methylation patterns between both promoters (CAG reprogramming vs. CAG puromycin). DNA methylation analysis was performed by bisulfite conversion using primers designed to specifically amplify the promoter driving the expression of the reprogramming 6-factor cassette (CAG reprogramming). Another primer pair was designed to amplify the puromycin resistance promoter (CAG puromycin). Additionally, primers non-selectively amplifying both promoters were used (CAG) (Fig. 3a). The three amplicons were sequenced with two different oligonucleotides, i.e., S2 (Fig. 3) and S1 (Supplementary Fig. 6). Fibroblasts and Rh_ESCs were included as negative controls.

Three teratomas and three iPSC lines were included in the analysis. The methylation of the reprogramming cassette promoter (CAG reprogramming, 60–95% methylation) was higher than the methylation of the puromycin promoter (CAG puromycin, 17–30% methylation) in all samples (Fig. 3b,c) (Supplementary Fig. 6a,b). As expected, the non-selective analysis of both promoters (CAG) showed intermediate values. No significant differences were found between teratomas and iPSC for any of the two promoters. These findings suggest that epigenetic modifications are mainly triggered already during reprogramming of fibroblasts into iPSCs and not during differentiation of the iPSC during the teratoma formation. GpC island-specific evaluation of the bulk analysis of the samples shows homogenous methylation levels at all CpG sites in all amplicons (Fig. 3e). This advocates against high heterogeneity in the epigenetic modifications in the different GpC sites contained in each promoter. The two different primers used for sequencing show consistent results (Fig. 3) (Supplementary Fig. 6). This data shows that already iPSCs have close to maximum methylation levels of the CAG reprogramming similar to the teratomas. This may explain the down-regulation of the expression of the exogenous pluripotency factors as shown by IHC of the teratomas. However, the CAG puromycin promoter is significantly less methylated than the CAG reprogramming promoter demonstrating differential methylation of the two sequence-wise identical CAG promoters present in the *piggyBac* construct.

### Transposon removal by re-expression of *pBase-tdTomato* in iPSCs

**Figure 4:**
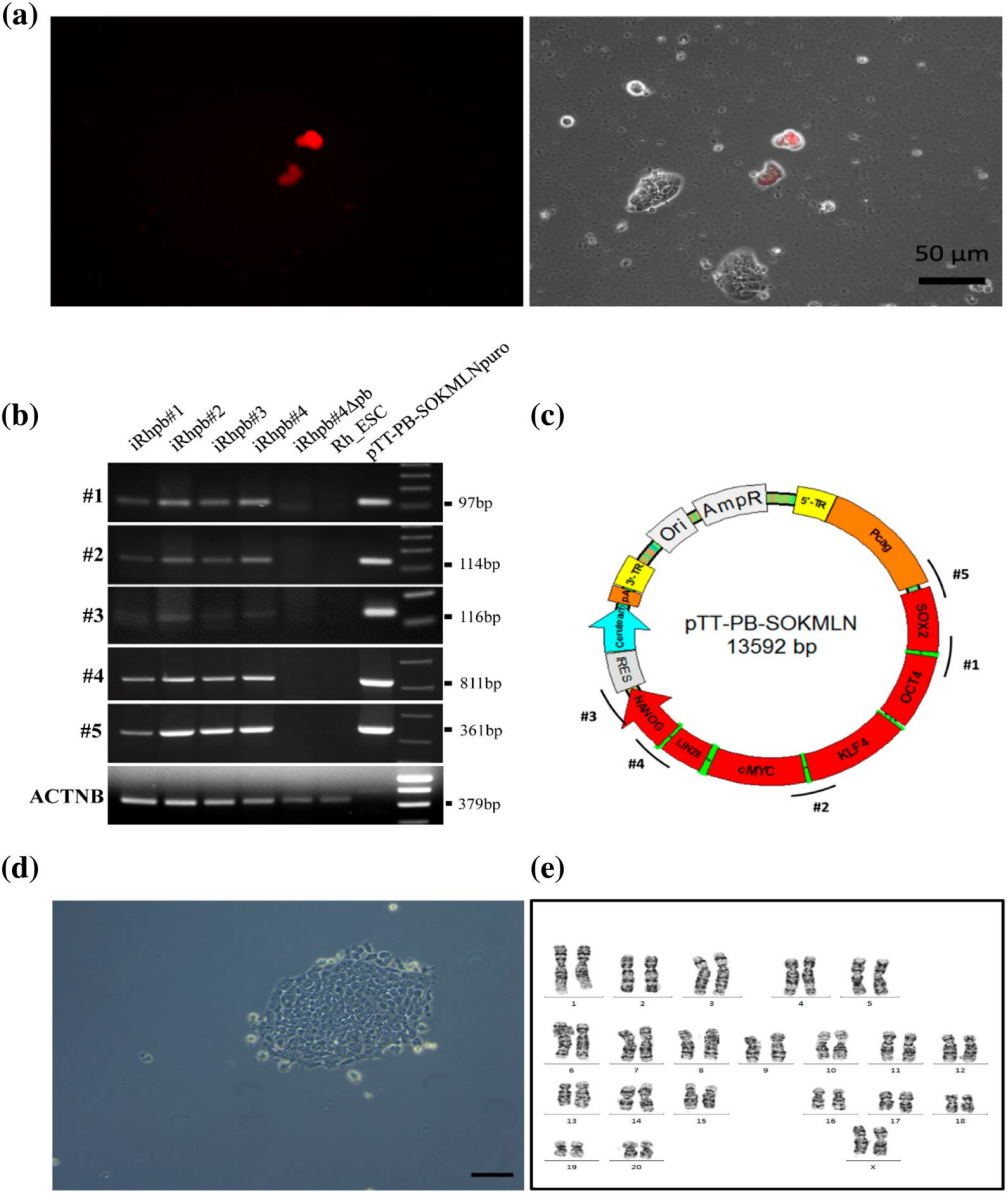
Transposon removal by re-expression of *pBase-tdTomato* in rhesus iPSC lines. (**a**) iRhpb#4 two days after transfection with the transposase vector *pBase-tdTomato*. Left, red fluorescence channel; right, merged brightfield/fluorescence. tdTomato positive clones were sorted and isolated in order to check for the removal of the transposon (Scale bar 50 µm). (**b**, **c**) Transposase transfected clones were screened for the presence/absence of the *piggyBac* construct by PCR. (**b**) iRhpb#4Δpb shows no evidence of the presence of the exogenous construct. Beta-actin (*ACTNB*) was used as control for the presence of gDNA. Positive controls used for the analysis were iRhpb#1, iRhpb#2, iRhpb#3, and *pTT-PB-SOKMLNpuro* plasmid DNA. Rh_ESCs were used as negative control. Individual gels (#1–5 and *ACTNB*) were assembled to obtain (**b**). The individual gels are separated by white horizontal lines. All samples within one gel were run in one PCR. (**c**) PCR amplicons for the detection of the reprogramming vector are schematically represented (#1–5). (**d**) Brightfield image of iRhpb#4∆pb (Scale bar 50 µm). (**e**) Representative G-banded karyotype of iRhpb#4∆pb (passage 42). Karyotyping yielded a normal female rhesus macaque chromosome set without any numerical or structural abnormality.

We have shown high methylation of the CAG reprogramming promoter (Fig. 3) (Supplementary Fig. 6). However, RT-PCR analysis still detected the transposon-encoded transcripts in the iPSC lines (Supplementary Fig. 3). In order to generate a transgene-free iPSC line and demonstrate that the stability of the iPSC line is based exclusively on endogenous gene expression, we exemplarily tried to remove the reprogramming transposon from DPZ_iRhpb#2 and 4. Cells were re-transfected with the transposase vector *pBase-tdTomato* (Fig. 4a) (Supplementary Fig. 7)^27^. Two days after transfection, clusters of Tomato-positive cells were identified. Tomato-positive cells were sorted by FACS to evaluate transfection efficiency and for selection of the potential transgene-free clones. Transfection efficiency was evaluated for DPZ_iRhpb# 2 and 4. The average percentage of tdTomato positive cells for the two iPSC lines was 11% (DPZ_iRhpb# 4) and 15% (DPZ_iRhpb# 2) (Supplementary Fig. 7b). For the selection of transgene-free clonal lines cells, different clones were sorted and analyzed for the presence/absence of the transposon in gDNA by PCR (Fig. 4b,c). Five different primer pairs were used to detect different fragments of the transposon (Fig. 4c). Successful removal of the transposon was only achieved for DPZ_iRhpb# 4. In this line, one out of 28 clones showed no presence of the reprogramming cassette (Fig. 4b).

The resulting transgene-free subline DPZ_iRhpb#4Δpb remained undifferentiated under feeder-free conditions (Fig. 4d, compare with Fig. 1c) and characterization was performed again after transposon removal. iRhpb#4∆pb showed alkaline phosphatase activity and expression of pluripotency markers OCT4A, LIN28, TRA-1-60, SOX2, TRA-1-81, and SALL4 on the protein level (Supplementary Fig. 8a,b). iRhpb#4∆pb also produced teratomas (Supplementary Fig. 8c). To confirm the absence of the transposon on the mRNA level, iRhpb#4∆pb was analyzed by RT-PCR. iRhpb#4∆pb showed no exogenous expression of the *LIN28-NANOG* fusion transcript, confirming absence of transposon-derived mRNAs. Additionally, iRhpb#4∆pb presents endogenous *OCT4A*, *SOX2*, *NANOG*, and *c-MYC* expression similar to iRhpb#1–4 (Supplementary Fig. 3). Importantly, no chromosomal rearrangements were detectable by karyotyping in iRhpb#4∆pb after excision of the *piggyBac* construct (Fig. 4e).

In summary, iRhpb#4∆pb cells remain pluripotent under feeder-free conditions and have a normal karyotype (Fig. 4e) after removal of the transposon.

### Derivation of clonal rhesus macaque pluripotent stem cell lines

To generate clonal iPSC lines with defined mutations, single-cell isolation and propagation protocols need to be established. Two cell lines, Rh_ESC and DPZ_iRhpb#4 were selected to develop a work-flow for clonal rhesus PSC line generation, based partially on the work-flow published by Chen and colleagues for human iPSC (Fig. 1c)^29^.

**Figure 5:**
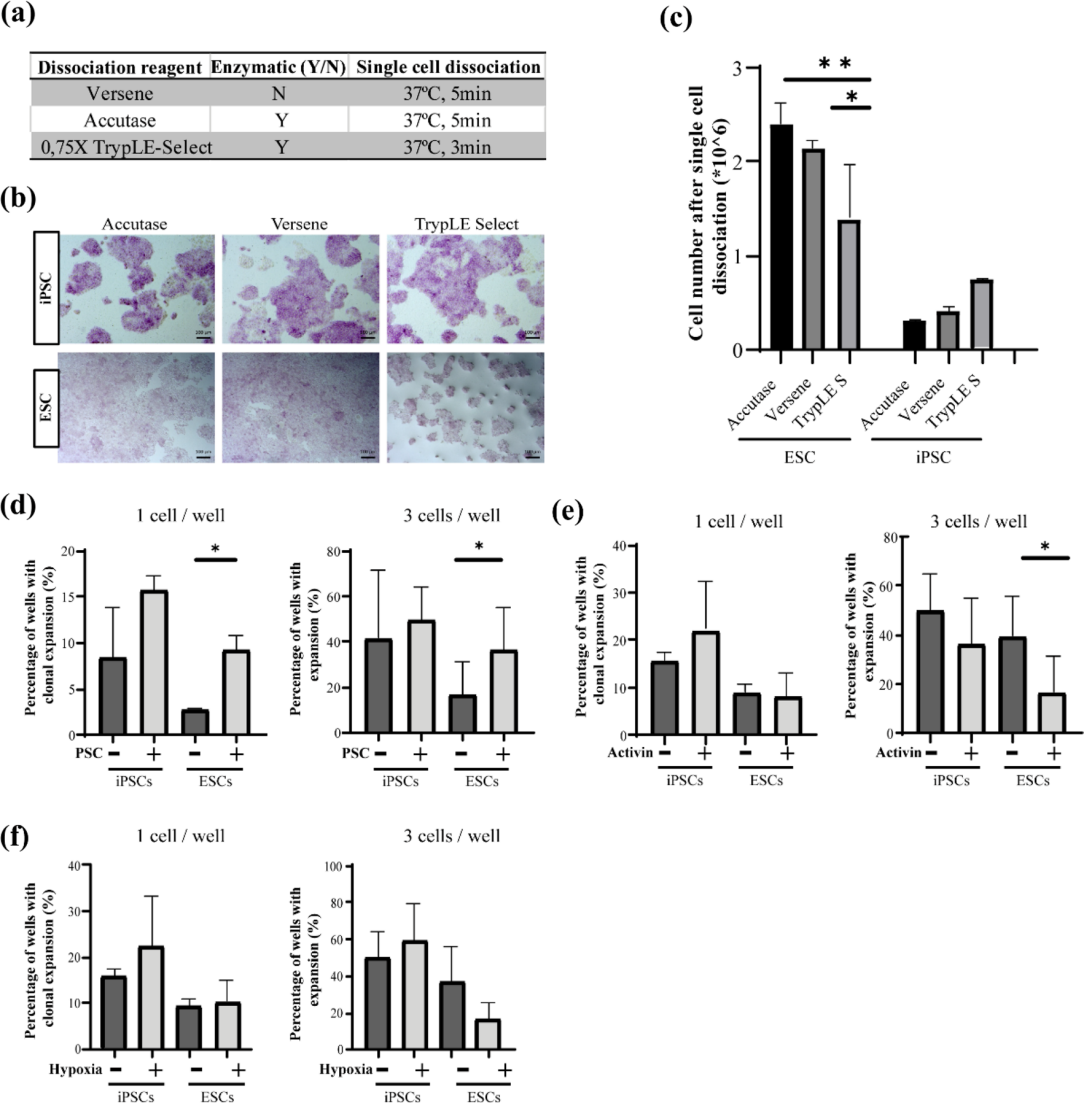
Clonal cell line derivation from rhesus macaque pluripotent stem cells. (**a**) Reagents and conditions tested for dissociation. (**b**) AP lkaline phosphata staining after single-cell dissociation and re-seeding of DPZ_iRhpb#4 and Rh_ESC (Scale bar 100 µm). (**c**) Cell numbers three days after single-cell dissociation of 2 × 10^5^ cells (n = 4) (Mean ± SD). (**d**–**f**) Percentage of cells (iRhpb#4 and Rh_ESC) achieving clonal expansion after dissociation and sorting in 96 well plates. Left graph, clonal expansion after sorting one cell per well. Right graph, colony expansion after sorting three cells per well (Mean ± SD). (**d**) Cells sorted in standard UPPS medium (Control) and in UPPS medium supplemented with 10 µM pro-survival compound (+ PSC). (**e**) Percentage of wells with clonal expansion of cells sorted in UPPS + PSC (Control) vs. cells sorted in UPPS + PSF + Activin (50 ng/µl). (**f**) Clonal expansion of cells sorted in UPPS + 10 µM PSC in normoxia and hypoxia (5% O_2_). (*) p < 0.05, (**) p < 0.01.

The first step towards single-cell cloning is reliable and complete dissociation of cell clusters to a single cell suspension. In order to find the most suitable dissociation reagent, we tested three reagents commonly used in human PSC protocols: versene, accutase, and TrypLE Select (Fig. 5a). After dissociation with the different reagents, we analyzed proliferation and differentiation of the cells. Five days after splitting, spontaneous differentiation of Rh_ESC and DPZ_iRhpb#4 was assessed by alkaline phosphatase staining and proliferation by cell counting. The starting cell number was 200.000 cells. No significant differentiation was found for any of the three tested reagents (Fig. 5b). Proliferation analysis of DPZ_iRhpb#4 revealed no significant differences between the dissociation reagents. In contrast, Rh_ESCs dissociated with accutase or versene showed higher proliferation recovery in comparison with TrypLE Select (Fig. 5c). These data suggest that accutase and versene may be more suitable reagents for single-cell dissociation compared to TrypLE for Rh_ESCs.

**Figure 6:**
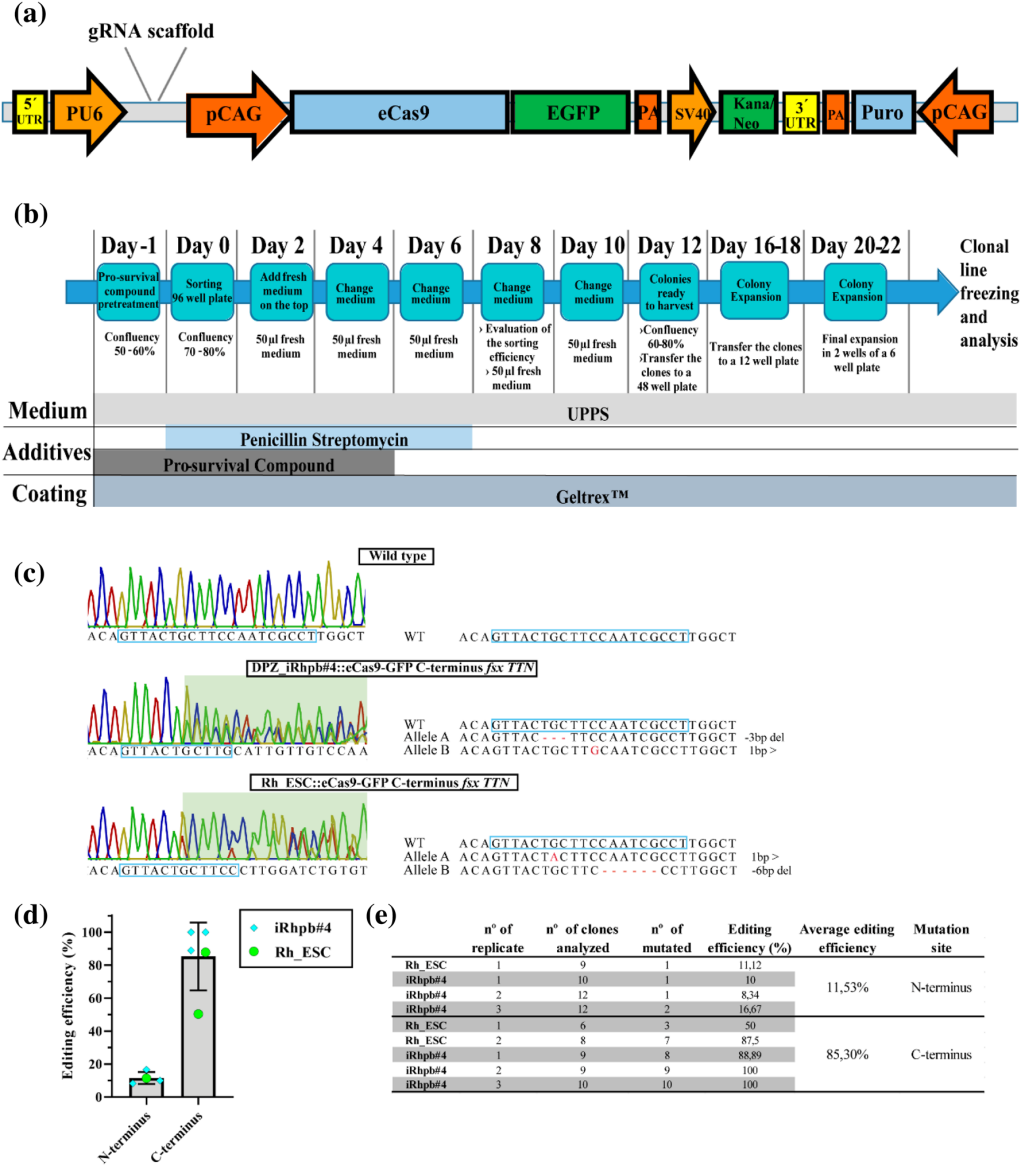
Genome editing of rhesus macaque pluripotent stem cells using the *piggyBac* system. (**a**) Schematic representation of the *piggyBac* vector used for CRISPR/Cas9 expression, *pTT-PB-pCAG-eCas9-GFP-U6-gRNA-Neo*. Three independent expression units are contained in the *piggyBac* construct. (1) U6 promoter regulating the expression of tRNA-gRNA, (2) CAG promoter-driven expression of eCas9-eGFP fusion transcript. A T2A sequence separates both coding sequences. (3) SV40 promoter driving the expression of the Kanamycin/Neomycin resistance gene. (**b**) Single-cell cloning work-flow. (**c**) Proof of concept mutation analysis of monoclonal lines by PCR amplification of the C-terminal *TTN* locus and Sanger sequencing. The figure shows the representative sequence analysis of two different clones, one of iRhpb#4 and the other Rh_ESC. Left figures show chromatograms of the wildtype *versus* the mutated lines. The mutation site is indicated by the appearance of doublet peaks and the gRNA binding site by a green rectangle. (**d**,**e**) Genome editing efficiency of the *TTN* regions encoding the N- and C-termini, respectively. Four (N-terminus) and five (C-terminus) independent experiments were performed. All transfections led to mutated clones with an efficiency ranging from 8.3 to 100%. Overall, mutation efficiency was 11.53% for the N-terminual gRNA and 85.3% for the C-terminual gRNA. (**d**) Graph representing editing efficiency (Mean ± SD). (**e**) Breakdown of the different experiments performed.

We then transfected Rh_ESC and DPZ_iRhpb#4 with the *piggyBac* vector *pTT-PB-pCAG-eCas9-GFP-U6-gRNA-Neo* together with *pBase-tdTomato* encoding the transposase, for constitutive expression of Cas9 in the PSC (Fig. 6a). In order to analyze the transfection efficiency, replicates for each one of the two PSC lines were sorted 5 days after transfection. The overall transfection efficiency for the combination of transposon/transposase CRISPR/Cas vector system was 49,03% for iRhpb#4 and 12% for Rh_ESC (Supplementary Fig. 9). For the derivation of the monoclonal lines containing the mutations of interest, we decided to passage the cells three times before sorting in order to increase the likelihood that the GFP-positive population integrated the transposon. Single cells were sorted into a 96-well plate by FACS. For control purposes, one or three cells were sorted into each well. This approach allowed us to expand clones and to obtain a representative number for analysis. We tested three different conditions to improve clonal expansion (Fig. 6b). Firstly, culture with and without pro-survival compound, secondly, with and without Activin A and, thirdly, under normoxia and hypoxia (5% O_2_). Only pro-survival compound significantly increased cell survival and/or proliferation (Fig. 5d), while neither Activin supplementation for two days after sorting (Fig. 5e) nor hypoxia (Fig. 5f) improved clonal expansion. In summary, the use of accutase in combination with medium supplemented with pro-survival compound for clonal expansion provides optimized conditions for the establishment of clonal rhesus PSC lines.

### Genome editing of rhesus macaque stem cells using the *piggyBac* transposon Cas9/GFP vector

In order to validate the clonal expansion protocol, we designed and tested CRISPR/Cas9 guides to target clinically relevant mutation sites in the sarcomeric gene Titin (*TTN*). Two sites were targeted, one encoding the N-terminus and another one encoding the C-terminal part of the protein (rhesus exons homologous to human *TTN* exons 38 and 280, respectively; ENST00000589042.5). Guides were inserted in the *pTT-PB-pCAG-eCas9-GFP-U6-gRNA-Neo* vector and the construct was transfected together with the transposase-expressing vector following a DNA-integrative approach. After transfection, PSC were passaged three times before the generation of the monoclonal lines. Increasing the time span (8–10 days/three passages) between transfections and sorting we expect that the GFP signal comes from constitutive and not transient expression of the construct to ensure the integration of the transposon. Even though transfection efficiency following the protocol detailed in the methods part is usually high, variation between different cell lines was observed (Supplementary Fig. 9b). The *pTT-PB-pCAG-eCas9-GFP-U6-gRNA-Neo* vector contains a neomycin resistance cassette that can be used to enrich the transfected population before sorting (Fig. 6a). In parallel to the transfection of the PSC for sorting, we performed bulk population analysis of the targeted *TTN* sites in fibroblasts and iPSC. We performed Sanger sequencing (data not shown) and T7 endonuclease I assay^35^ in order to gain insights into the functionality of the gRNAs plus vector before starting the laborious single-cell cloning process (Supplementary Fig. 10). Both gRNAs showed evidence of on-target editing activity (Supplementary Fig. 10). While the targeted site encoding the N-terminal sequence showed only faint digestion products in the T7 endonuclease I assay, the targeted site encoding the C-terminal sequence showed more intense bands. After transfection and expansion of the transfected cells, the single-cell cloning protocol developed above was followed, and monoclonal lines containing frameshift mutations (*fxs*) in both *loci* were expanded. Eight clones with truncating N-terminal mutations were obtained: three for Rh_ESC and five for DPZ_iRhpb#4. Mutations in the different clones were analyzed by PCR amplification of the targeted genomic *loci* and subsequent sequencing of the products (data not shown). The efficiency of the process was also analysed for the C-terminus, and independent biological replicas for each cell line were performed (transfection, sorting, and expansion). For the C-terminal mutation, 37 mutated clones were obtained from 42 clones analyzed in total (DPZ_iRhpb#4: 27 mutated/28 analyzed; Rh_ESC: 10 mutated/14 analyzed). Exemplary chromatograms are shown in Fig. 6c for DPZ_iRhpb#4::eCas9-GFP C-terminus *fsx TTN* and Rh_ESC::eCas9-GFP C-terminus *fsx TTN*. The overall efficiency of the process for this gRNA was 85% (mutated clones/analyzed clones; Fig. 6d,e). The N-terminal gRNA worked less efficiently than the C-terminal gRNA. From 49 clones analyzed 5 mutated were identified, with an overall efficiency of 11.53% (Fig. 6d,e). However, each experiment performed with any of the gRNAs led to at least one mutated clone (number of clones analyzed per experiment ranges between 9 and 12, Fig. 6e). In conclusion, we established tools and a protocol for the robust and reliable generation of genetically modified clonal rhesus macaque ESC and iPSC lines carrying clinically relevant mutations.

### Isogenic control cell line generation and characterization

The next step after the generation of the mutated clones is to assess the phenotypic alterations in differentiated cells generated from the pluripotent stem cells lines, e.g., iPSC-derived cardiomyocytes^33^. Downstream analysis of the mutated cells requires the generation of proper isogenic controls, to exclude possible effects of the cloning process and the genome editing construct expression. Therefore, isogenic controls were generated for both lines. Transfection of the cells with the *piggyBac* Cas9 vector without guides (*pTT-PB-pCAG-eCas9-GFP-U6-gRNA-Neo*), but with transposase, was performed (DPZ_iRhpb#4::eCas9-GFP and Rh_ESC::eCas9-GFP). Isolation of appropriate isogenic clonal control populations expressing the Cas9-GFP construct was achieved for both Rh-ESC and DPZ_iRhpb#4 (Supplementary Fig. 11a). Expression of Cas9-GFP was evaluated every passage by fluorescent microscopy and at passage 5 by immunostaining (Supplementary Fig. 11b). All cells in the populations show Cas9-GFP expression (Supplementary Fig. 11a,b).

In order to show the functionality of the clonal lines (mutated and isogenic controls) derived from the genome editing process, basic characterization of Rh_ESC and DPZ_iRhpb#4 sublines was performed. Isogenic controls (Supplementary Fig. 11c) and *TTN* mutated lines still express key pluripotency markers (Supplementary Figs. 11c, 12a). Additionally, all lines present a normal chromosome number (Supplementary Fig. 12b, shown for DPZ_iRhpb#4::eCas9-GFP C-terminus *fsx TTN*). Finally, in order to ensure that the cloning process does not affect the differentiation potential of the iPSCs we performed spontaneous in vitro differentiation (shown for DPZ_iRhpb#4::eCas9-GFP and Rh_ESC::eCas9-GFP). During the embryoid body formation, no down-regulation of the GFP expression in the cell aggregates was observed (Supplementary Fig. 11d). Additionally, immunostaining of both cell lines analyzed showed differentiation into representative cell types of the three germ layers (Supplementary Fig. 11e,f). These results indicate that the cloning protocol neither changed the cells' differentiation potential nor caused major chromosomal abnormalities in the genome-edited PSCs.

## Discussion

NHPs as our closest phylogenetic relatives are excellent animal models to study human diseases. The phylogenetic relationship is reflected in similar physiology, genetics, life span, and relatively equal size^5,6,8–10,13^. In order to model human pathologies with genetic origin in NHPs, one of the main strategies is to edit the embryonic genome. Therefore, genome editing tools for these applications need to be validated in vitro before in vivo translation to guarantee the efficiency, accuracy and hence safety of the process. NHP-PSCs share significant parts of their molecular signatures with those of the pluripotent cells present in the early embryo. Therefore, testing of novel editing tools in PSCs will help to assess their performance in vivo^7,36,37^.

Four novel rhesus macaque iPSC lines were generated using our previously published 6-reprogramming-factor transposon^27^. The generated iPSCs were pluripotent as indicated by pluripotency marker expression and teratoma formation. RT-PCR was performed with primer pairs able to discriminate between endogenous and exogenous origin of pluripotency-related genes. Robust expression of endogenous *OCT4A, SOX2, NANOG*, and *c-MYC* transcripts was detected in the iPSCs as judged by comparison with Rh_ESCs. However, the analysis also revealed the presence of *piggyBac*-derived exogenous transcripts showing that the expression cassette was not fully silenced in iPSCs. The potency of the novel NHP-iPSC lines was also evaluated by teratoma formation. All lines analyzed formed teratomas. We were wondering whether the *piggyBac* cassette would be silenced during iPSC generation or upon iPSC differentiation over several weeks in the context of teratoma development. Interestingly, most teratoma sections were negative for the pluripotency markers OCT4A, LIN28, TRA-1-60 and NANOG, for which we have established specific and sensitive immunohistochemical detection protocols^34,38^. We found only scattered and small clusters of pluripotency factor-positive cells. This indicates down-regulation of endogenous pluripotency genes and, even more important, also down-regulation of the reprogramming factor expression from the reprogramming construct in the differentiated cells of the teratoma.

In order to get first insights into *piggyBac* silencing in the teratoma, we performed comparative methylation analysis of the two CAG promoters present in the *piggyBac* construct isolated from iPSCs and the teratomas. There was no clear difference between the methylation patterns found in iPSCs and in teratomas. However, between the two promoters, i.e., the CAG puromycin and the CAG reprogramming, we found differential methylation patterns. Surprisingly, the CAG reprogramming was highly methylated (in most samples close to 100%) in comparison with the CAG puromycin promoter, which shows around 30% methylation in teratomas and around 20% in iPSCs. These findings were generally reproduced in all three independent iPSC lines. This suggests that the cells can discriminate between the two CAG promoters in the construct, which are sequence-wise identical. Either the gene products (indirectly) regulate the methylation of the promoters or the broader DNA sequence context in which the actual CAG promoters are embedded. Targeted methylation has been previously described for this and other reprogramming approaches^39^. Moreover, the high methylation of the CAG reprogramming was not only found in the teratomas, but also in the iPSC. This indicates that methylation of the reprogramming construct occurs mainly during fibroblast reprogramming and/or early passages of the iPSCs, but not during differentiation, as we initially hypothesized. Constitutive reprogramming factor expression could lead to differentiation failure and tumorigenicity^40,41^. However, in our experiments, we observed mainly differentiated cells within the teratomas. Even more important, also the iPSC line after deletion of the *piggyBac* cassette (see below) shows some remaining OCT4A-positive cells in the teratoma, indicating that not (only) the exogenous OCT4A expression was still detectable in the teratomas, but also the endogenous protein.

The *piggyBac* transposon can be removed from the genome of the cells without leaving a footprint^31,32,42,43^. Even though the teratoma showed silencing of the exogenous reprogramming factors in most of the cells, this would not be a safe condition regarding cell transplantation, and exogenous genetic material present in the iPSC genome is not acceptable for transplantation purposes. In order to generate transgene-free macaque iPSC, we exemplarily excised the reprogramming transposon from the genome of one iPSC line. The resulting transgene-free clone was then fully re-characterized. Pluripotency factor expression and potency of the cell line remained unchanged after transposon removal. In addition, karyotyping showed that the excision-ligation process did not generate any detectable chromosomal abnormalities. We are aware of reprogramming approaches based on non-integrating vectors like Sendai viruses and episomes^33^. However, we have shown that rhesus monkey iPSC generation is significantly less efficient than human iPSC generation^33^. Considering this, the integrating, yet reversible *piggyBac* approach for NHP-iPSC generation is still useful because of its robustness^43^. In fact, *piggyBac*-based reprogramming proved useful for the generation of marmoset monkey^27^, baboon^44^ and rhesus monkey (present study) iPSCs. From the latter two species we obtained iPSCs also from adult and aged animals.

We adapted three of the generated NHP-iPSC lines to feeder-free conditions. Feeder-free culture is essential to obtain pure iPSC populations without the presence of other (feeder) cells. Furthermore, this allows up-scaled production and facilitates more efficient use of biotechnological tools, like CRISPR/Cas9^45,46^. The cell lines remained undifferentiated under the new conditions. Importantly, also the transgene-free iPSC line was cultured in feeder-free conditions. Hence, feeder-free and transgene-free culture of *piggyBac*-derived rhesus iPSCs is possible.

Genome editing and single-cell cloning of PSCs is challenging due to the low efficiency of the process and stress-induced death of the stem cells forced to single-cell separation^28,29^. Fine-tuning of the protocols developed for human^29,47^ and mouse PSCs^48^ was required in order to generate a robust protocol for rhesus macaque PSCs^49^. We have demonstrated that accutase and versene are more suitable dissociation reagents for the generation of rhesus ESC single-cell suspensions than TrypLE. Furthermore, we tested different compounds and conditions to increase the number of surviving single cells/clones after sorting. ROCK inhibitor (pro-survival compound, PSC) shows a beneficial effect in single-cell survival in the two lines analyzed, while hypoxia and Activin showed no effect on the cloning efficiency in our experimental setup. Taking these results together, we developed a robust single-cell cloning protocol for rhesus PSC. Finally, we used a *piggyBac* vector containing eCas9-GFP plus guide RNAs to target two clinically relevant loci in the *TTN* gene. The *piggyBac* transposon combining all required components of the CRISPR/Cas9 editing machinery in one vector in combination with the fine-tuned protocol for single-cell cloning allowed us to reach high efficiency in the generation of mutated clones, e.g. over 85% for the C-terminal *TTN* mutation.

Altogether, we demonstrated the suitability of the *piggyBac* system to reprogram and gene edit rhesus macaque iPSCs. We developed an efficient platform to evaluate CRISPR/Cas-based genome editing approaches in PSCs before in vivo application. In combination with the new protocol for clonal expansion of gene-edited rhesus PSCs, this represents a useful in vitro screening platform for gene editing and thereby contributes to the 3Rs (reduce, replace, and refine) in animal experimentation. Differential methylation of the two identical CAG promoters present in the vector was encountered, with high methylation of the CAG reprogramming and low methylation of the CAG puromycin promoter. This may suggest a specific methylation response to the gene product controlled by the respective promoters and deserves further investigations. Finally, we have demonstrated removal of the reprogramming transposon by re-expression of the transposase, resulting in transgene-free clonal iPSCs cultured under feeder-free conditions. However, a higher efficiency of the removal procedure of the *piggyBac* transposon would be desirably.

## Materials and methods

### Animals

The German Primate Center (DPZ) is registered and authorized by the local and regional veterinary governmental authorities. Rhesus macaque skin samples were made available during necropsy from animals kept in the context of an unrelated project (Approval number of the Niedersächsisches Landesamt für Lebenmittelsicherehit und Verbraucherschutz 33.42502-04-16/2370). All animal experiments and methods were performed in accordance with relevant guidelines and regulations for animal use, and the institutional guidelines of the DPZ for the care and use of rhesus macaques were followed. All methods were carried out in accordance with the arrive guidelines. The cell lines DPZ_iRhpb#1–3 iPSC were derived from an adult male macaque (16 years) and the cell line DPZ_iRhpb#4 iPSC from a female (8 years). Regarding the use of mice in the context of the mouse embryonic fibroblast generation and the teratoma formation experiments, please see “Mouse embryonic fibroblasts (MEFs)” and “Teratoma formation and histological analysis”, respectively.

### Isolation of rhesus macaque primary fibroblasts

Isolation of rhesus macaque fibroblasts from skin and gingiva biopsies was performed according to Ref.^44^. In brief, a tissue biopsy of approximately 1 × 1 cm was washed with PBS [1% (v/v) Penicillin/Streptomycin (Gibco), 0.25 μg/mL Amphotericin B (Sigma)] and subcutaneous adipose tissue was removed with a scalpel. Clean dermal tissue was chopped with scissors into small pieces and incubated in Collagenase type IV solution (10 mg/mL in DMEM) (Gibco) for three hours at 37 °C rotating (80 rpm). After digestion, cells were pelleted by centrifugation (5 min, 300 g, RT). Fibroblasts were seeded in 10 cm diameter gelatine-coated culture dishes (0,1% gelatine; Sigma) and cultured in Rh15 medium [DMEM (Gibco), 15% (v/v) Fetal Bovine Serum (Gibco), 1% (v/v) Penicillin/Streptomycin (Gibco), 0,1% (v/v) Amphotericin B (Sigma), 1% (v/v) MEM Non-Essential Amino Acids Solution (Gibco), 2 mM GlutaMAX (Gibco) 10 ng/mL FGF (ThermoFisher)] at 37 °C and 5% CO_2_. Accutase dissociation reagent (Gibco) was used for passaging.

### Mouse embryonic fibroblasts (MEFs)

Gamma-irradiated MEFs, derived from E12.5 embryos of CD1 mice, were used as feeder cells. The generation was described previously^27^.

### Nucleofection and reprogramming procedure

Primary fibroblasts were reprogrammed using the 6-factors-in-one-vector *piggyBac* construct, coding for the marmoset monkey reprogramming factors SOX2, OCT4, KLF4, c-MYC, NANOG, and LIN28. 1 × 10^6^ fibroblasts were transfected with a 4D-nucleofector device (program CA-137; Lonza), using P2 nucleofection solution. The reprogramming transposon vector was co-transfected with *pBase-tdTomato* transposase vector (9 µg, and 6 µg pDNA, respectively). The efficiency of the nucleofection was estimated two days after transfection by the expression of the Tomato reporter from the transposase vector. Two days after transfection antibiotic selection was started adding 1.5 µg puromycin (Sigma) to the fibroblast culture medium. Five days after selection the fibroblasts were plated (0.2 × 10^5^ cells per plate) on gelatine coated 10 cm plates with MEFs and cultured in embryonic stem cell medium (ESM) [KO-DMEM (Gibco), 20% (v/v) KnockOut Serum Replacement (Gibco), 1% (v/v) Pen/Strep (Gibco), 0,25 μg/mL Amphotericin B (Sigma), 1% (v/v) MEM (Gibco), 2 mM GlutaMAX (Gibco), 50 μM 2-mercaptoethanol (Gibco), 10 ng/mL FGF (ThermoFisher)]. ESM was supplemented with 2 mM valproic acid (Calbiochem) for the first 6 days of culture. 20 days after transfection first colonies were identified and new colonies appeared until day 60. Colonies were manually picked and transferred to new plates with MEFs. Around passage 5, mass passage was possible using Collagenase type IV (1 mg/mL). Rhesus macaque iPSC long-term storage was done using Rh_freezing medium [ESM, 20%DMSO (Sigma), 10% FBS (Gibco)] at − 150 °C.

### Adaptation to feeder-free culture conditions

Rhesus iPSC (iRhpb#4) in passage 10 were manually picked and transferred into 6 cm plates coated with Geltrex (Thermo Fisher Scientific). Cells were cultured according to our previously published protocol^33^. NHP pluripotent stem cells medium UPPS [StemMACS™ iPS-Brew XF, human—Stem cell media (Miltenyi Biotec) supplemented with IWRI (1 µM) (Sigma), and Chir (0.5 µM) (Merck/Millipore)]. The first day after picking, the medium was supplemented with ROCK inhibitor, PSF (Pro-survival Compound, 5 µM) (Calbiochem). After 3–5 passages, the culture stabilized showing no morphological differentiation. After 5 passages cells were split using versene-EDTA solution (Thermo Fisher) (split ratios used were 1/10 to 1/15). To freeze rhesus iPS, the freezing medium was used (Essential 8 medium, 20% DMSO (Sigma), and 10 µM PSF).

### Alkaline phosphatase

Alkaline phosphatase activity was demonstrated using Leukocyte Alkaline Phosphatase Kit (Sigma), following manufacturer’s recommendations.

### Immunofluorescence

Rhesus iPSCs were cultured in wells equipped with coverslips. When the colonies reached 30–50% confluence, they were fixed with 4% PFA (v/v) for 20 min (RT). Fixation solution was removed and cells washed three more times with PBS. 1% BSA in PBS was used for blocking. For intracellular epitopes 1% BSA, TritonX-100 (0.1%, Sigma) was used. Primary antibodies were diluted in PBS/1% BSA and incubated for 1 h at 37 °C. Subsequently, cells were washed with PBS and incubated with Alexa 488/555/594-coupled secondary antibodies (Life Technologies) diluted in PBS/1% (w/v) BSA. Finally, coverslips were incubated with PBS/DAPI solution (0.5 µg/mL), for 10 min. After staining the coverslips were removed from the cell culture wells, and mounted using Citifluor mountant medium (CITIFLUOR ltd.). Immunofluorescence images were taken with a Zeiss Observer Z1 (Zeiss). Primary antibodies used were OCT4A (Cell Signalling OCT-4A C52G3, 1:1600), SOX2 (Cell Signalling C70B1, 1:200), NANOG (Cell Signalling D73G4, 1:400), TRA-1-60 (eBioscience 14-8863, 1:100), TRA-1-81 (eBioscience 14-8883, 1:100), LIN28 (Cell Signalling A177, 1:100), SALL4 (Abcam ab57577, 1:200), Cas9 (Merck Millipore MAC133, 1:200), AFP (Sigma A8452, 1:100), β-Tubulin III (Sigma, T8660, 1:500), smooth muscle actin (Sigma A2547, 1:500). Secondary antibodies Alexa-488/555/594 conjugated Ab Donkey anti-Mouse IgG (H + L) (Life technologies A21202, 1:1000), Donkey anti-Rabbit IgG (H + L) (Invitrogen A-21206, 1:1000) and Goat anti-mouse IgM (H + L) (Invitrogen A-21042, 1:1000).

### Reverse transcription PCR

RNA was extracted from cell pellets using RNAeasy Mini Kit (Qiagen). Contaminating genomic DNA was eliminated by treating the samples with RNase-free DNase (Qiagen). cDNA was synthesized from 1 µg RNA using Oligo(dT) primers and the Omniscript RT Kit (Qiagen), according to manufacturer instructions. Primer pairs (Sigma) were designed and used for detection of the different pluripotency factors, and to discriminate between the endogenous (*OCT4A, SOX2, NANOG*, and *c-MYC*) and the exogenous (*LIN28-NANOG fusion*) transcripts (Supplementary Table 1). As positive and negative controls rhesus embryonic stem cells (Rh_ESC) and MEFs, respectively, were used. Endogenous beta-actin (*ACTNB*) expression was used as a house-keeping gene. Taq DNA Polymerase with Standard Taq Buffer (New England BioLabs) was used for all RT-PCRs performed. Original gel electrophoresis pictures of RT-PCR analysis can be found in Supplementary Fig. 13.

### Teratoma formation and histological analysis

Animal experiments in the context of the teratoma assay were approved by the Niedersächsisches Landesamt für Lebenmittelsicherehit und Verbraucherschutz under the licence number 33.42502-04-113/09. For teratoma formation assay, 8*10^5^ cells from each iPSC line and 2 × 10^5^ MEFs were co-injected subcutaneously into male immunodeficient RAG2^−/−^γc^−/−^ mice. Before injection, cells were resuspended in PBS supplemented with Geltrex (ThermoFisher, 0.1 mg/mL), with a final injected volume of 120 µL. The development of the teratomas was regularly monitored by palpation, and as soon as teratomas reached a size of 5 to max. 10 mm, the mice were euthanized by cervical dislocation by qualified and authorized personnel following institutional and governmental guidelines. Teratomas were prepared and fixed as previously described^50^. Histological tissue sections were stained for the detection of representative markers of each germ layer. Primary antibodies used were β-Tubulin III (Sigma, T8660, 1:600), smooth muscle actin (SMA; Sigma, A2547, 1:1000) and SOX9 (Millipore, AB5535, 1:1000) for detection of ectoderm, mesoderm, and endodermal epithelium, respectively. Additionally, OCT4A (Cell Signalling OCT4-A C52G3, 1:1000), NANOG (Cell Signalling D73G4, 1:400), TRA-1-60 (eBioscience 14-8863, 1:50) and LIN28 (Cell Signalling A177, 1:100) primary antibodies were used for the detection of undifferentiated pluripotent cells in the teratomas.

### In vitro differentiation

Rhesus macaque iPSC were differentiated in vitro by embryoid body (EB) formation (according to Ref.^33^). In brief, iPSCs were digested with collagenase type IV (Worthington, 200 U/mL) for 10 min at 37 °C, washed and detached using a cell scraper (Sarstedt). Cell aggregates were cultures in UPPS medium for 24 h and transferred to differentiation medium (79 mL IMDM (Thermo Fisher), 20 mL FBS, 1 mL 100 × NEAA, 450 µM 1-thioglycerol (Sigma-Aldrich)). During the differentiation in suspension, medium was changed every second day. On day 8, EBs were transferred onto 0.1% gelatine-coated plates with coverslips and culture until day 25. At the end of the protocol, cells were fixed in 4% PFA.

### Promoter methylation assay

Promoter methylation analysis was performed in three different iPSC (DPZ_iRhpb#1–3) at different passages and in three teratomas (DPZ_iRhpb#1–3). As negative controls, Rh_ESC and rhesus macaque fibroblasts were included in the analysis. DNA methylation analysis of the CAG promoter was performed by bisulfite pyrosequencing. 500 ng of the isolated genomic DNA was used for the DNA bisulfite conversion reaction with the EZ DNA Methylation-Direct™ Kit (Zymo), according to manufacturer’s instructions.

For PCR, Fast Start Taq polymerase (Roche) was used to amplify the bisulfite-converted DNA. PCR conditions and primers were designed with the Pyrosequencing Assay Design Software (Qiagen). The *piggyBac* 6-factor-reprogramming construct contains two CAG promoters, one driving the expression of the reprogramming cassette, and the other one the puromycin resistance gene. In order to analyze both promoters separately, one assay for each CAG region was designed. Both assays contained the same forward primer but a different reverse primer located in the unique sequence of the reprogramming cassette (CAG Reprog) and the puromycin resistance gene (CAG Puro). In addition, a third assay was designed to evaluate both CAG promoters (CAG). For reducing the amplification bias, all three measurements were arranged in a nested/semi-nested approach consisting of two following PCRs for each assay. The first PCR round generates three different outer amplicons related to the three different assays (CAG Reprog, CAG Puro and CAG). In the second PCR round, one primer combination of two primers located within the CAG promoter sequence was applied to all three different outer amplicons (CAG Reprog, CAG Puro and CAG) and generate the same inner amplicon for all three assays. This inner amplicon was sequenced with two sequencing primers, which together covered 15 CpGs in total (S1: 7 CpGs; S2: 8 CpGs). As cycler conditions of the outer-PCR reactions, the standard PCR procedure according to manufacturer’s instruction was applied with a cycle number of 30 (annealing CAG Reprog: 60 °C; CAG Puro: 64 °C; CAG: 52 °C). The inner-PCR contained for all three assays the same combination of two primers. The cycler conditions of the inner-PCR corresponded to the standard PCR procedure according to manufacturer’s instruction (annealing 53 °C and 35 cycles). Finally, the products of the inner-PCR were sequenced in triplicates by pyrosequencing following the manufacturer’s instruction. 10 µL of the generated inner-PCR products were used for the immobilization to Streptavidin Sepharose HP beads (GE Healthcare Life Sciences). The pyrosequencing was performed on a Pyromark Q96MD system (Qiagen) and analyzed with the Pyromark Q-CpG software.

### *PiggyBac* transposon removal/rhesus iPSC transfection

iRhpb#4 in feeder-free conditions was nucleofected using 4D-nucleofector device (Lonza). 1 × 10^6^ cells were transfected with *pBase-tdTomato* transposase vector (6 µg), using P3 nucleofection solution and program CA-137 (Lonza). UPPS medium was supplemented for 2 days with PSF (5 µM) after transfection. After 3 days Tomato positive clones were sorted using SH800S Cell Sorter (Sony Biotechnology), sorting 10 cells per well in a 24 well plate. Potential transgene-free colonies were picked and expanded for 2–5 passages. Colonies were then pelleted and gDNA was extracted. Detection PCR was performed using primers specifically designed to detect different regions of the transposon (Supplementary Table 1). PCR was performed using Taq DNA polymerase with Standard Taq Buffer (New England BioLabs) according to manufacturer instructions. Original gel electrophoresis pictures of PCR analysis can be found in Supplementary Fig. 14. Transgene-free clones were checked for the absence of the reprogramming construct every 10 passages.

Transfection efficiency was evaluated 3 days after transfection by FACS sorting. The analysis was performed in triplicates for two iPSC lines in feeder-free conditions (iRhpb#2 and 4). 10 × 10^3^ events were recorded for the analysis of each sample.

### Karyotyping

Metaphase arrest, fixation, G banding, and analysis protocols were described in detail in Ref.^44^.

### *piggyBac* CRISPR/Cas vector generation

The *pTT-PB-pCAG-eCas9-GFP-U6-gRNA-Neo* vector was derived by modification of plasmid *pCAG-eCAS9-GFP-U6-gRNA*, which was a gift from Jizhong Zou (Addgene plasmid # 79145; http://n2t.net/addgene:79145; RRID: Addgene_79145). In order to convert this plasmid into a *piggyBac* transposon, the 5′- and 3′-PB inverted repeats were amplified from *pTT-PB-SOKMLN-Puro* by PCR and inserted respectively into the *Pac*I and *Not*I/*Sbf*I restriction sites using standard molecular cloning techniques. Additionally, a neomycin-resistance cassette was inserted into the *BsrG*I restriction site in order to enable antibiotic selection. Oligo sequences coding for the different gRNAs were cloned into the vector after linearization with *Bbs*I.

### Dissociation reagent testing

Two stem cell lines, iRhpb#4 and Rh_ESC, were dissociated with three different reagents. Dissociation conditions are listed in detail in Fig. 6A. After dissociation, cells were collected and centrifuged (180*g*, 5 min, RT). After centrifugation 200,000 cells were transferred into a well of a 6-well plate and cultured for 5 days. Alkaline phosphatase staining and cell counting were subsequently performed. Statistical analysis of the cell numbers was performed using GraphPad PRISM and one way ANOVA test [(*) p < 0.05, (**) p < 0.01.].

### T7 endonuclease I assay

T7 endonuclease I assay^35^ was performed according to manufactures instructions (New England Biolabs, M0302L). In brief, CRISPR target sites were amplified from the genomic DNA of the transfected cells. PCR products were purified, heated to 95 °C and re-hybridized. Finally, T7 endonuclease I was added to the re-annealed PCR products. Cleavage of the PCR product by the endonuclease due to the presence of DNA mismatches was evaluated in 1.5% agarose gels. Original gel electrophoresis pictures of T7 endonuclease I assay can be found in Supplementary Fig. 15.

### Clonal expansion analysis

iRhpb#4 and Rh_ESC were used for the testing of the clonal expansion potential after single-cell dissociation and FAC-sorting. The transfection efficiency of *pTT-PB-pCAG-eCas9-GFP* and *pBase-tdTomato* was evaluated three days after nucleofection according to the protocol detailed in “Promoter methylation assay” (“Materials and methods”). For single cell cloning, cells transfected with *pTT-PB-pCAG-eCas9-GFP-U6-gRNA-Neo* and *pBase-tdTomato* were cultured for three passages and digested with accutase (37 °C, 5 min). The SH800S Cell Sorter (Sony Biotechnology) was used to sort Cas9-GFP positive cells. To evaluate the effect of PSF during clonal expansion, a control group was sorted with no PSF and compared to a test group with PSF. For the evaluation of the effect of Activin A (50 µg/µL; Miltenyi), or hypoxia (5% O_2_), the different experimental groups were compared to each other. Since PSF turned out in the first experiment to support clonal cell line derivation, it was the standard based on which the remaining variables (Activin and hypoxia) were tested. All experiments were performed in triplicates. For the significance analysis, paired T-test was performed using GraphPad PRISM software.

### Induction of truncation mutations in rhesus macaque stem cells

Two different guide RNAs for the CRISPR/Cas9 system were cloned and validated in the *pTT-PB-pCAG-eCas9-GFP-U6-gRNA-Neo* vector (Supplementary Table 1). Transfection of the iPSC was described above (*piggyBac* transposon removal/rhesus iPSC transfection). Transfection efficiency of the *pTT-PB-pCAG-eCas9-GFP-U6-gRNA-Neo* vector was evaluated 5 days after transfection by FACS sorting (according to 4.12 *PiggyBac* transposon removal/rhesus iPSC transfection). After transfection rhesus iPSCs and ESCs were culture for three passages with/without Geneticin (Gibco) (200 µg/mL). Antibiotic selection was used depending on the proportion of GFP-positive cells in the transfected population. If the percentage of GFP positive cells was estimated (by fluorescence microscopy) lower than 10%, Geneticin was added to the medium. After expansion the cells were FACS-sorted in order to obtain clonal sublines, expanded, and pelleted for gDNA extraction and analysis (Macherey–Nagel™ NucleoSpin™ Gel and PCR Clean-up Kit). Primers for the mutation site amplification and PCR conditions are described in Supplementary Table 1. PCR products of the different clones were purified and sent for sequencing (LGC Genomics). Sequence analysis was performed using Serial Cloner, Geneious and Chromas software. Mutations in the two different alleles of the target gene of each clone were predicted using Poly Peak Parser^51^.

## Supplementary Information

Supplementary Information.
