# Supplementary material for: A piggyBac-based platform for genome editing and clonal rhesus macaque iPSC line derivation
Source: Sci Rep. 2021 Jul 29;11:15439. doi: 10.1038/s41598-021-94419-7 (PMC8322147; doi:10.1038/s41598-021-94419-7)
Supplement: Supplementary file 1 — Supplementary Information. [file 41598_2021_94419_MOESM1_ESM.docx]

**Supplementary material**

**A *piggyBac* based platform for genome editing and clonal rhesus macaque iPSC line derivation**

Ignacio Rodriguez-Polo^1,2^, Sophie Mißbach^1,2^, Stoyan Petkov^1,2^, Felix Mattern^3^, Anna Maierhofer^3^, Iga Grządzielewska^1,4^, Yuliia Tereshchenko^1,4^, Daniel Urrutia-Cabrera^1 #^, Thomas Haaf^3^, Ralf Dressel^2,5^, Iris Bartels^6^, Rüsdiger Behr^1,2^*

^1^ Research Platform Degenerative Diseases, German Primate Center – Leibniz Institute for Primate Research, Kellnerweg 4, 37077 Göttingen, Germany

^2^ German Center for Cardiovascular Research (DZHK), Partner site Göttingen, Germany

^3^Institut für Humangenetik, Universität Würzburg, Biozentrum, Am Hubland, 97074 Würzburg

^4^Max Planck molecular Biology program (M.Sc./Ph.D.), Justus-von-Liebig-Weg 11, 37077 Göttingen, Germany

^5^Institute of Cellular and Molecular Immunology, University Medical Center Göttingen, Humboldtalle 34, 37073 Göttingen, Germany

^6^Institute of Human Genetics, University Medical Center Göttingen, Robert-Koch-Str. 40, 37075 Göttingen, Germany

^#^Current address: Cellular reprogramming unit, Center for eye research Australia, 75 Commercial Road, Melbourne 3004, Australia

*Correspondence:

Rüdiger Behr

Research Platform Degenerative Diseases

German Primate Center – Leibniz Institute for Primate Research

Kellnerweg 4, 37077 Göttingen, Germany

Fax: 0049-(0)551-3851431

Email: rbehr@dpz.eu

|  | **Oligo name** | **Sequence** | **Tm (°C)** | **Elong (sec)** | **Amp (bp)** |
| --- | --- | --- | --- | --- | --- |
| RT-PCR | G302_LIN28-NANOG_fw | AGCCATATGGTAGCCTCATGTCC | 62 | 55 | 811 |
|  | G70_LIN28-NANOG_rev | GGTTGCTCCAGGTTGAATTGC |  |  |  |
|  | G2212_OCT4 endo_fw | GAGAAGGAGAAGCTGGAGCAA | 52,9 | 60 | 841 |
|  | G2213_OCT4 endo_rev | ACATCCTTCTCGAGCCCAA |  |  |  |
|  | G2008_SOX2 endo_fw | GGTAGGAGCTTTGCAGGAAGT | 61 | 30 | 428 |
|  | G2009_SOX2 endo_rev | CCAACGATGTCAACCTGCATG |  |  |  |
|  | G2237_NANOG endo_fw | CAGAGATACCTCAGCCTCCAG | 54,4 | 35 | 562 |
|  | G2238_NANOG endo_rev | CTTCAGGTTGCATGTTCGT |  |  |  |
|  | G2010_cMYCendo_fw | CTGGTACTCCATGAGGAGACA | 61 | 60 | 715 |
|  | G2011_cMYCendo_rev | CTCAGCCAAGGTTGTGAGGTT |  |  |  |
|  | G2204_beta actin | GGTAGTTTCGTGGATGCCACA | 61 | 30 | 379 |
|  | G2205_beta actin | GACCTGACTGACTACCTCATG |  |  |  |
| *PiggyBac* detection | G1842_piggydetecF2Afw | GTGAAACAGACTTTGAATTTTGACC | 54 | 10 | 97 |
|  | G1843_piggydetecF2Arev | AATCCGAAGCCAGGTGTC |  |  |  |
|  | G1844_piggydetecE2Afw | TACACATGAAGAGGCATTTTCAATG | 55 | 10 | 114 |
|  | G1845_piggydetecE2Arev | TGCTGAAGCTGACGTTGA |  |  |  |
|  | G1846_piggydetecpAfw | TGCCACTCCCACTGTCCTTTCCTA | 57 | 10 | 116 |
|  | G1847_piggydetecpArev | CAATCCTCCCCCTTGCTG |  |  |  |
|  | G302_LIN28-NANOGfw | AGCCATATGGTAGCCTCATGTCC | 62 | 55 | 811 |
|  | G70_LIN28-NANOG_rev | GGTTGCTCCAGGTTGAATTGC |  |  |  |
|  | G244_Pcag-SOx2fw | GGGGACGGCTGCCTTCGG | 60 | 20 | 361 |
|  | G395_Pcag-SOx2rev | CGGTCGGGGCTGTTCTTCTG |  |  |  |
|  | G2204_beta actin | GGTAGTTTCGTGGATGCCACA | 61 | 30 | 379 |
|  | G2205_beta actin | GACCTGACTGACTACCTCATG |  |  |  |
| gRNAs | G1806_TTN_N-terminusfw | CACCgGCTGACTACACCTTTGTGGC |  |  |  |
|  | G1807_TTN_N-terminusrev | AAACGCCACAAAGGTGTAGTCAGCc |  |  |  |
|  | G1822_TTN_C-terminusfw | CACCgGTTACTGCTTCCAATCGCCT |  |  |  |
|  | G1823_TTN_C-terminusrev | AAACAGGCGATTGGAAGCAGTAACc |  |  |  |
| Clonal line analysis | G2616_N-termfw | GCATGGTGGCACAGAGTTGT | 61 | 100 | 1861 |
|  | G2617_N-termrev | TAGACAGCTGCTAGGGACAC |  |  |  |
|  | G2686_N-termfw2 | GCACTAGTATCTCCACCTGG | 61 | 60 | 825 |
|  | G2687_N-termrev2 | GCAACCACCAATCTACATCTC |  |  |  |
|  | G2654_C-termfw | GCGACTCTGCCCAACTACAT | 61 | 60 | 828 |
|  | G2655_C-termrev | GCAGTGTTGGTGACTTCCTC |  |  |  |
| Methylation analysis | G2656_CAG_Reprogfw | GGGATTTTTTTTGTTTTAAATTTGTG | 60 | 25 | 401 |
|  | G2657_CAG_Reprogrev | AATTCCACCACACTAAACTAAT |  |  |  |
|  | G2658_CAG_Purofw | GGGATTTTTTTTGTTTTAAATTTGTG | 64 | 35 | 575 |
|  | G2659_CAG_Purorev | TATCACCCTCTCAATATACCTATC |  |  |  |
|  | G2660_CAGfw | GTAGTTATTGTTTTTTATGGTAA | 52 | 25 | 373 |
|  | G2661_CAGrev | TAATAAAACAACACAATAACCAACAC |  |  |  |
|  | G2662_CAG_Inner_fw | GGGATTTTTTTTGTTTTAAATTTGTG | 53 | 29 | 333 |
|  | G2663_CAG_Inner_rev | TAATAAAACAACACAATAACCAACAC |  |  |  |
|  | G2664_CAG_Inner_Seq_fw | CATAAACATAATTAACAAAAACTCT |  |  |  |
|  | G2665_CAG_Inner_Seq_rev | CCCCCCCCATTTCCTT |  |  |  |

**Suppl. Table 1:** Oligos used in this study. PCR conditions defined by annealing temperature (Tm), elongation (Elong), and amplicon (Amp). **(*)** 5‘-Biotin.


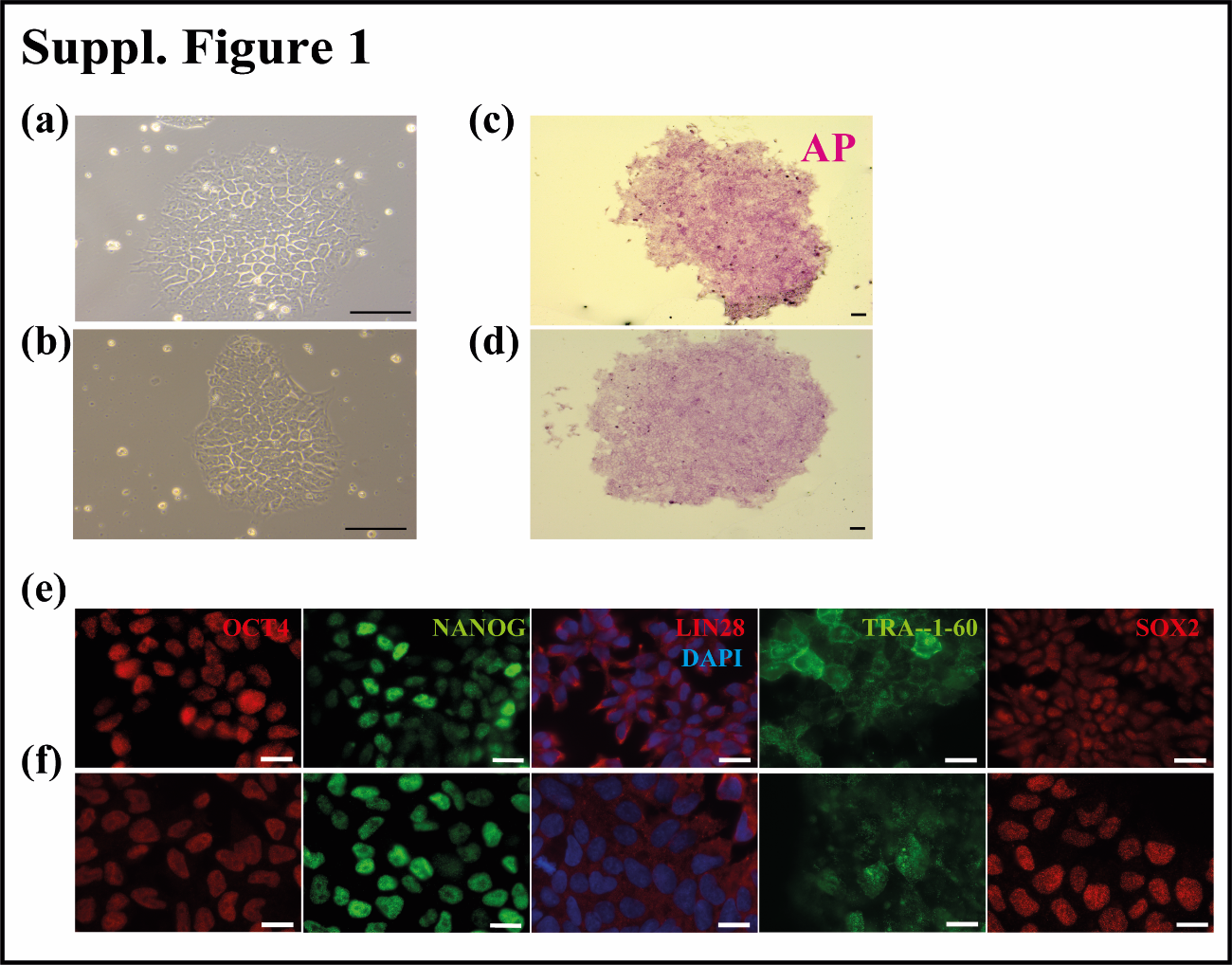


**Suppl. Figure 1:** Rhesus iPSC in feeder-free conditions. Bright-field pictures **(a)** and alkaline phosphatase staining (AP) **(c)** of iRhpb#2 and iRhpb#3 **(b)**, **(d)** (Scale bars 100µm). Immunofluorescence staining of DPZ_iRhpb#2 **(e)** and 3 **(f)** for the markers OCT4A (OCT4), NANOG, LIN28 (plus DAPI), TRA-1-60, SOX2. (Scale bar 20 µm).


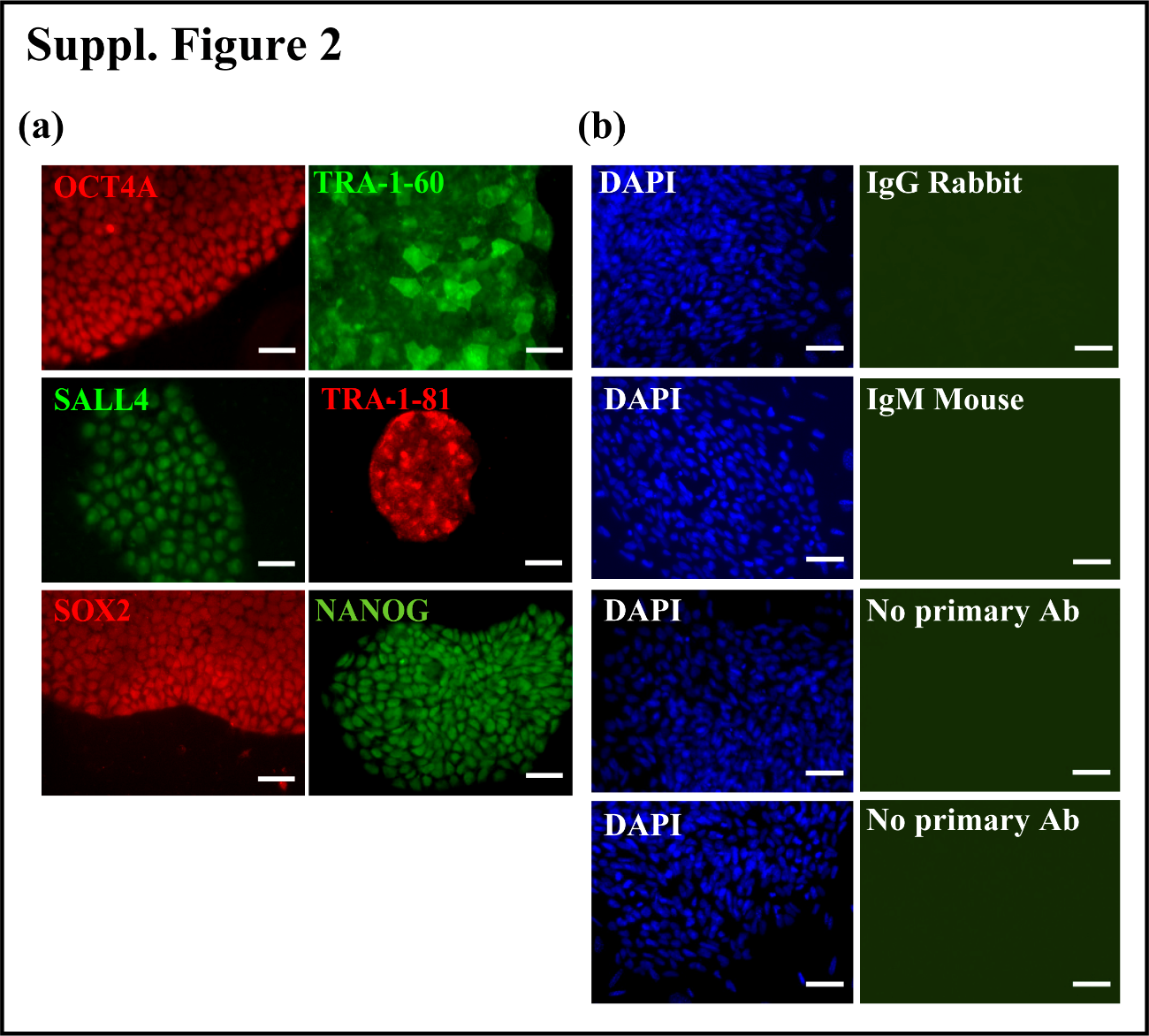


**Suppl. Figure 2:** Immunofluorescence staining of DPZ_iRhpb#4 and negative/isotype controls. **(a)** Immunofluorescence staining of DPZ_iRhpb#4. Detection of OCT4A, LIN28, TRA-1-60, SOX2, TRA-1-81, and SALL4. OCT4A, LIN28, and SOX2 expression come from both endogenous and *piggyBac* expression. (Scale bar 20 µm). **(b)** Immunofluorescence negative and isotype controls. Isotype controls. FITC (left column) plus DAPI (right column) (Scale bar 100 µm).


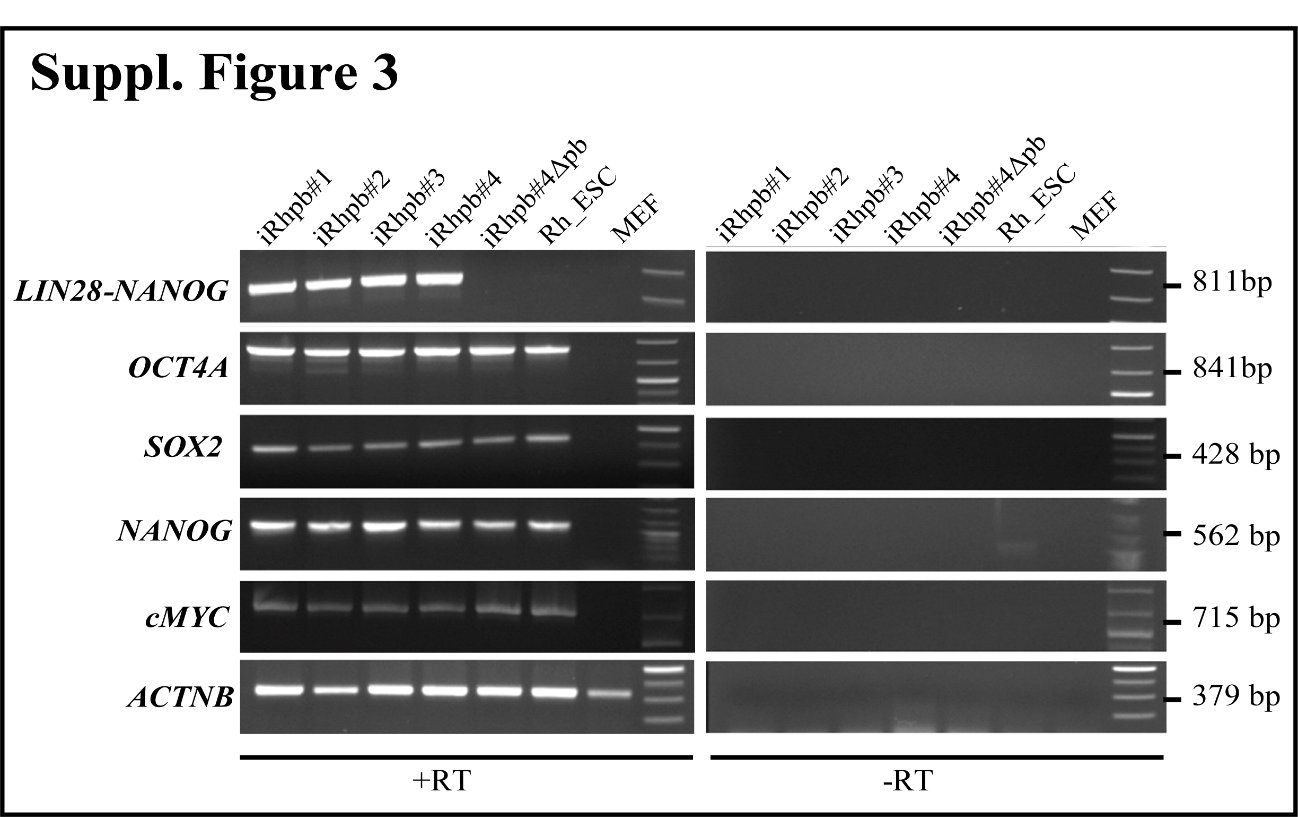


**Suppl. Figure 3:** Rhesus iPSC characterization by RT-PCR: Expression analysis of the four generated Rhesus iPSC lines (DPZ_iRhpb#1-4), and transgene-free DPZ_iRhpb#4Δpb. Rhesus embryonic stem cells (Rh_ESC) and MEFS were added as positive and negative controls, respectively. Primers for the RT-PCR were designed for the specific amplification of the endogenous pluripotency factors *OCT4A, SOX2, NANOG*, and *c-MYC. LIN28-NANOG* amplicon shows transposon expression in the transgenic lines and absence of exogenous transcript in DPZ_iRhpb#4Δpb. Beta-actin expression (*ACTNB*) was used as a housekeeping gene (marker 1kb Plus DNA ladder, NEB).


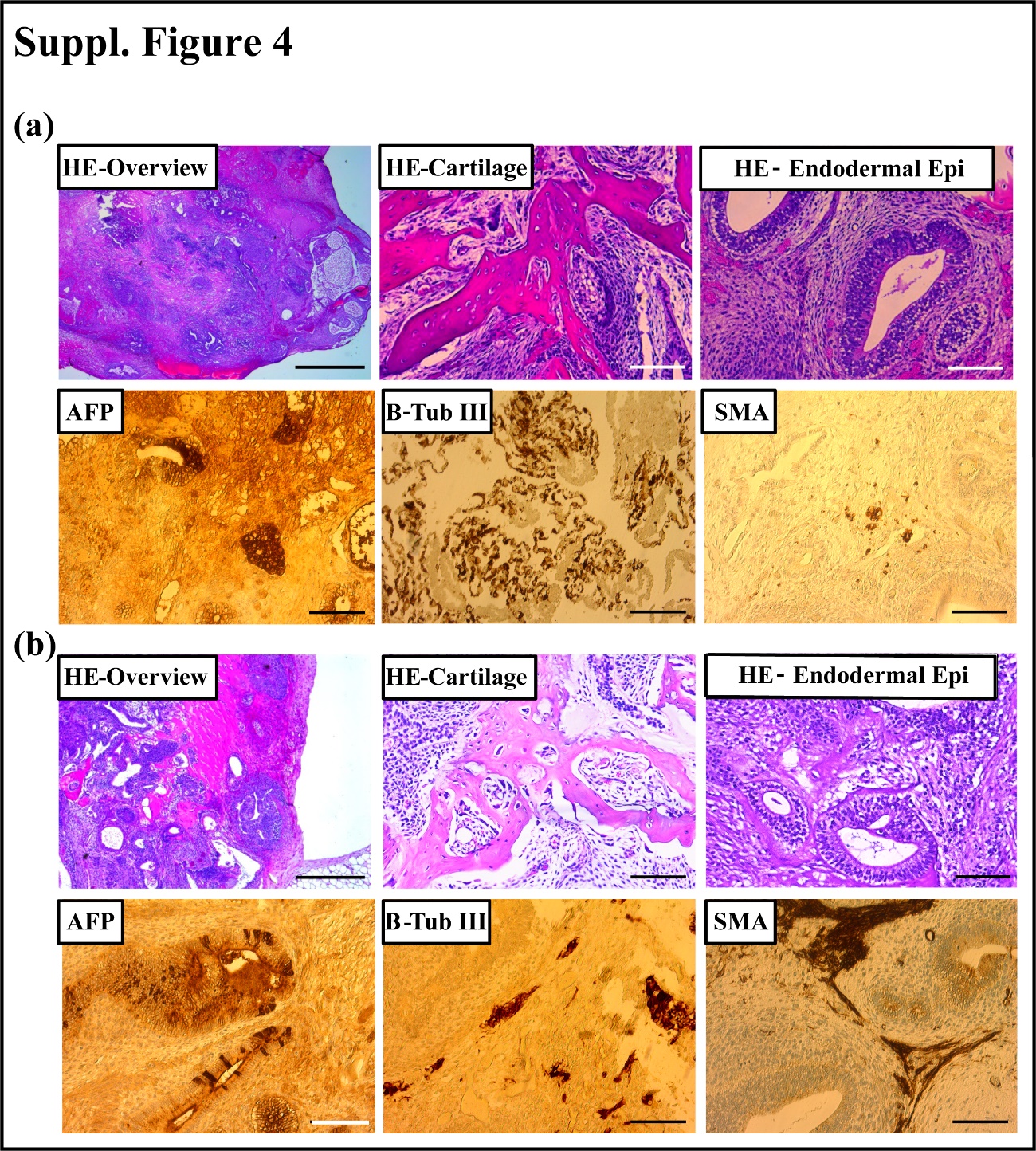


**Suppl. Figure 4:** Immunohistochemical analysis of DPZ_iRhpb#1 **(a)** and DPZ_iRhpb#3 **(b)** teratomas. Teratoma sections were stained for representative markers of the three germ layers: β- tubulin III, smooth muscle actin (SMA), and Alpha-1-Fetoprotein (AFP). Complementarily HE staining was performed to identify specific cytological features of representative tissues. β- Tubulin III indicate mesodermal differentiation. Smooth muscle actin and AFP staining show ectodermal and endodermal differentiation, respectively. Presence of cartilage indicates mesoderm. Moreover, gut endodermal epithelium support AFP staining, showing also the presence of endodermal tissue (Scale bar 100 µm).


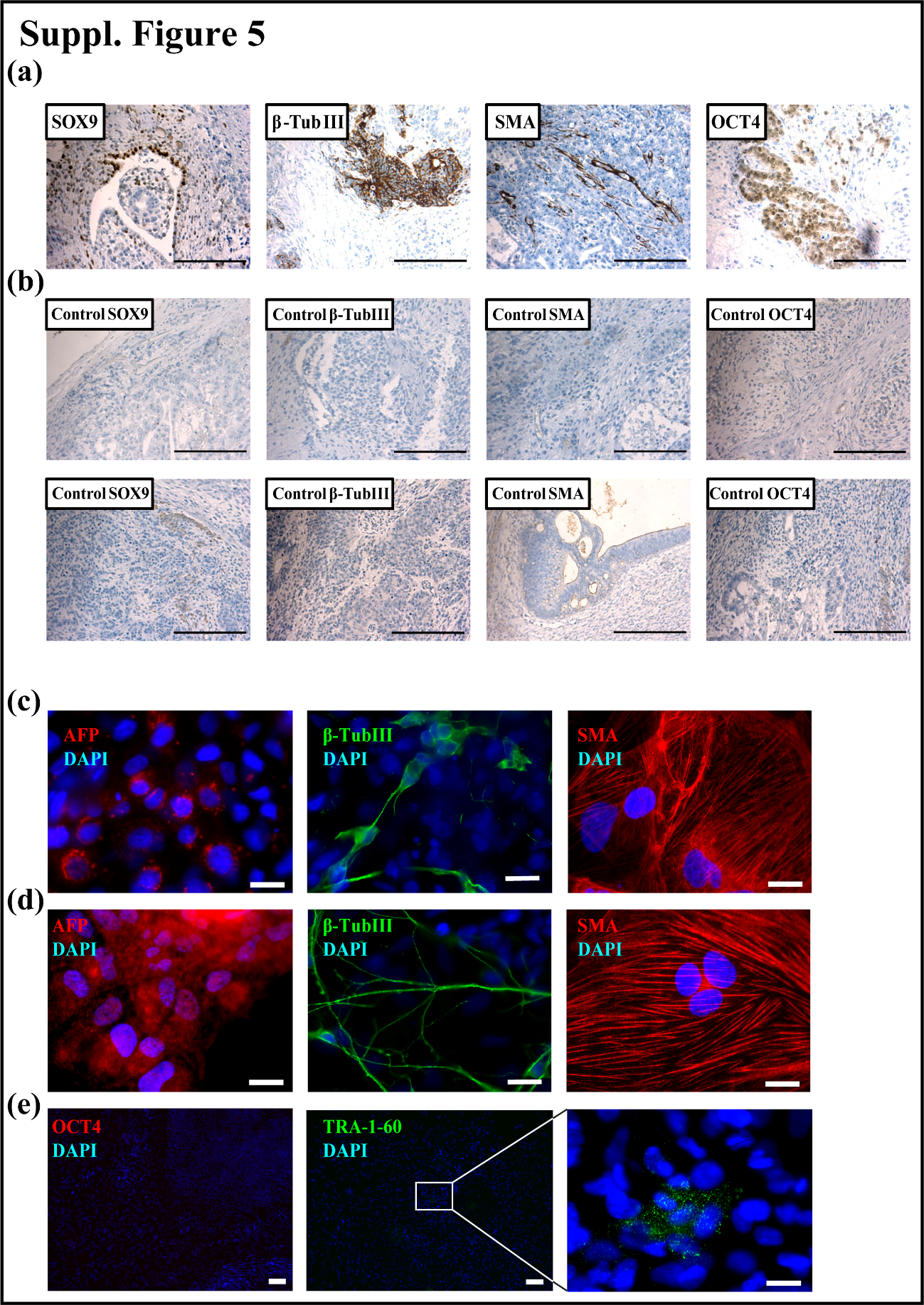


**Suppl. Figure 5:** Immunohistochemical analysis of *in vivo* differentiation of DPZ_iRhpb#4 (teratoma), isogenic controls (teratomas DPZ_iRhpb#1 and DPZ_iRhpb#3) and immunocytochemical analysis of *in vitro* differentiation of DPZ_iRhpb#3 and 4 in feeder-free conditions. **(a)** Teratoma sections from DPZ_iRhpb#4 stained for SOX9, β- tubulin III, smooth muscle actin (SMA), and OCT4A. (Scale bar 100 µm). **(b)** Isogenic controls for the teratoma staining. Upper row shows controls for DPZ_iRhpb#1, lower row the controls for DPZ_iRhpb#3. Isogenic controls for the markers SOX9, β- tubulin III, SMA, and OCT4A (Scale bar 100 µm). **(c) (d)** Staining for the markers α-fetoprotein (AFP), β- tubulin III and SMA of embryoid bodies (EB) generated from **(c)** DPZ_iRhpb#3 and **(d)** DPZ_iRhpb#4 iPSC in feeder-free conditions (Scale bar 20 µm). β-Tubulin III staining indicates ectodermal differentiation. Smooth muscle actin indicates mesodermal differentiation, and AFP staining demonstrates endodermal differentiation**. (e)** DPZ_iRhpb#3 EBs stained for OCT4A and TRA-1-60. OCT4A staining shows no signal (Scale bar 100 µm). TRA-1-60 staining shows a small cluster of cells that stain positive for the marker, middle picture is an overview and the right picture high magnification (Scale bars 100 / 20 µm).


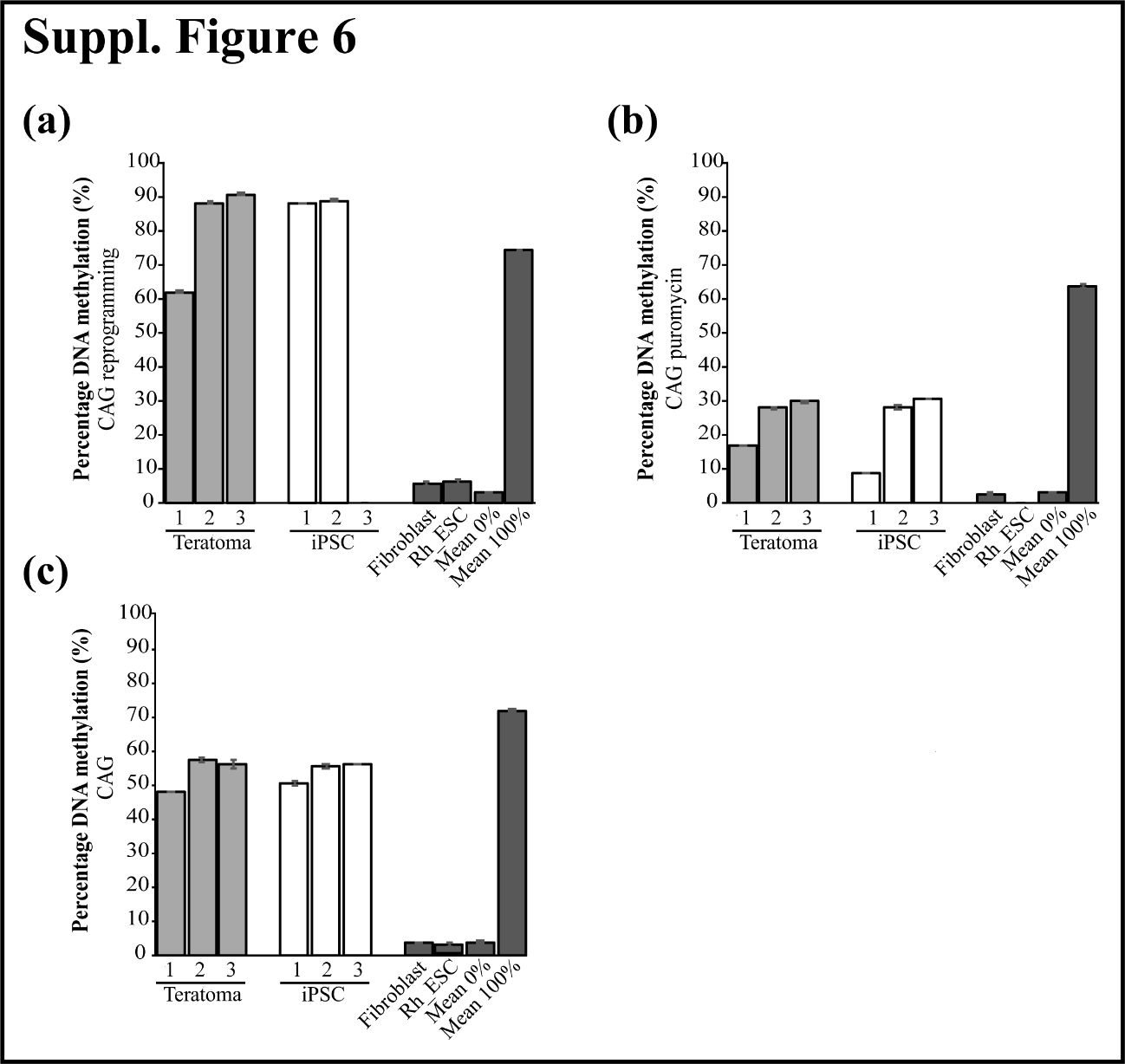


**Suppl. Figure 6:** Methylation analysis of the reprogramming construct using sequencing primer, S1, while data shown in main Figure 3 were generated using an alternative sequencing primer S2. Methylation analysis of three teratomas (teratomas 1-3), and three iPSC (iPSC 1-3). Two samples were included as negative control: Fibroblast and Rh_ESC. Internal controls are represented (Mean 0% and Mean 100%) (Mean±SD). **(a)** CAG reprogramming methylation analysis. **(b)** CAG puromycin methylation analysis. **(c)** CAG (including both promoters) methylation analysis.


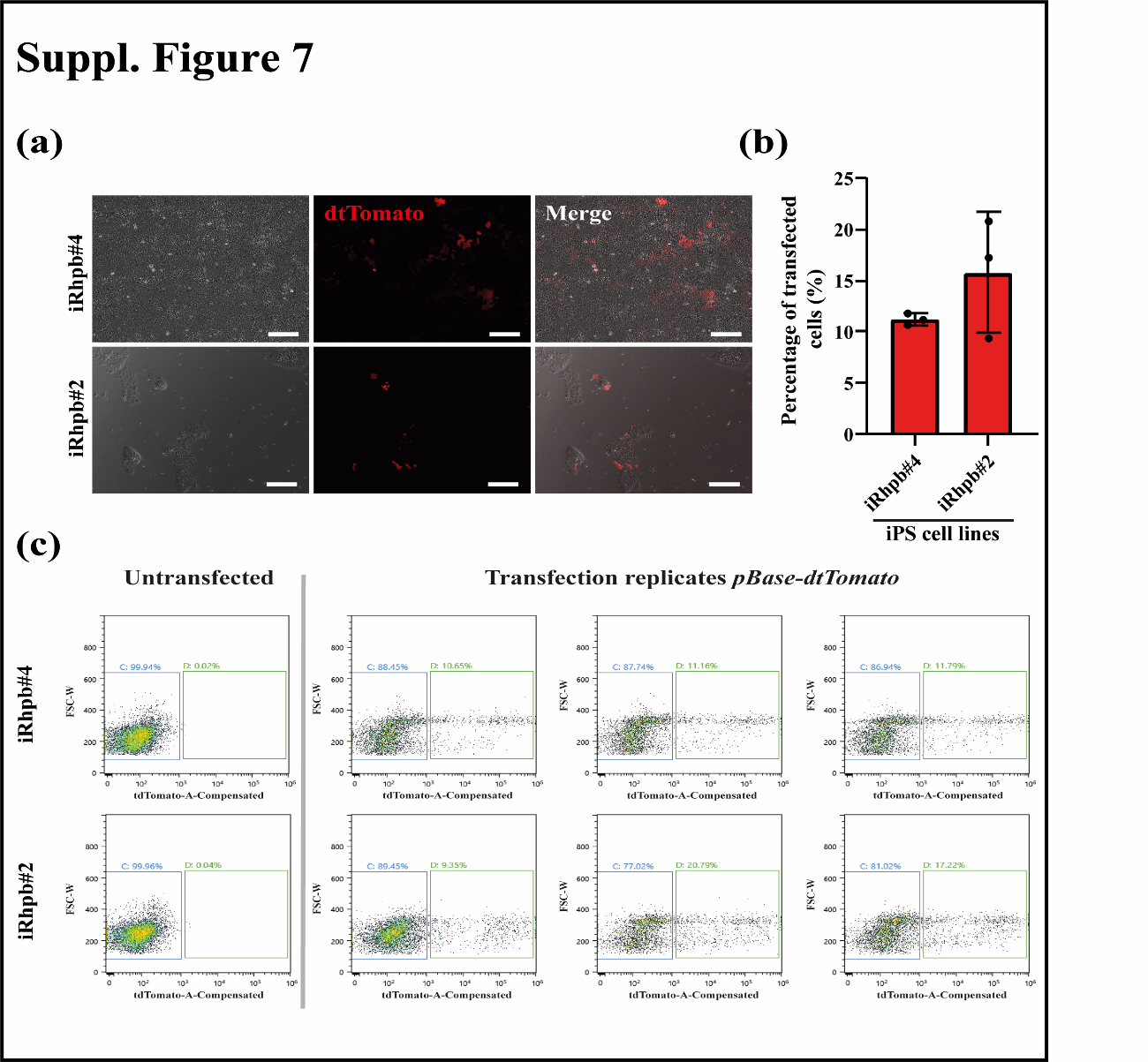


**Suppl. Figure 7:** Transfection efficiency of the *pBase-tdTomato* vector into rhesus iPSC. Efficiency was evaluated by fluorescence-activated cell sorting (FACS). **(a)** Representative images (bright-field, fluorescence, and merge) of transfections performed for each cell line included in the analysis (iRhpb#2 and 4) (day 3 after transfection) ((Scale bars 100 µm)). **(b) (c)** Three transfections per cell line were analyzed to calculate the percentage of transfected cells. FACS analysis was performed according to the expression of the fluorescent reporter in the transposase vector (tdTomato+ cells). **(b)** Graph representing the efficiency (Mean ±SD)(n=3+3). (c) Gating strategy used for sorting including as negative control, non-transfected cells (x-axis tdTomato Compensated) (y-axis. Forward scatter-width).


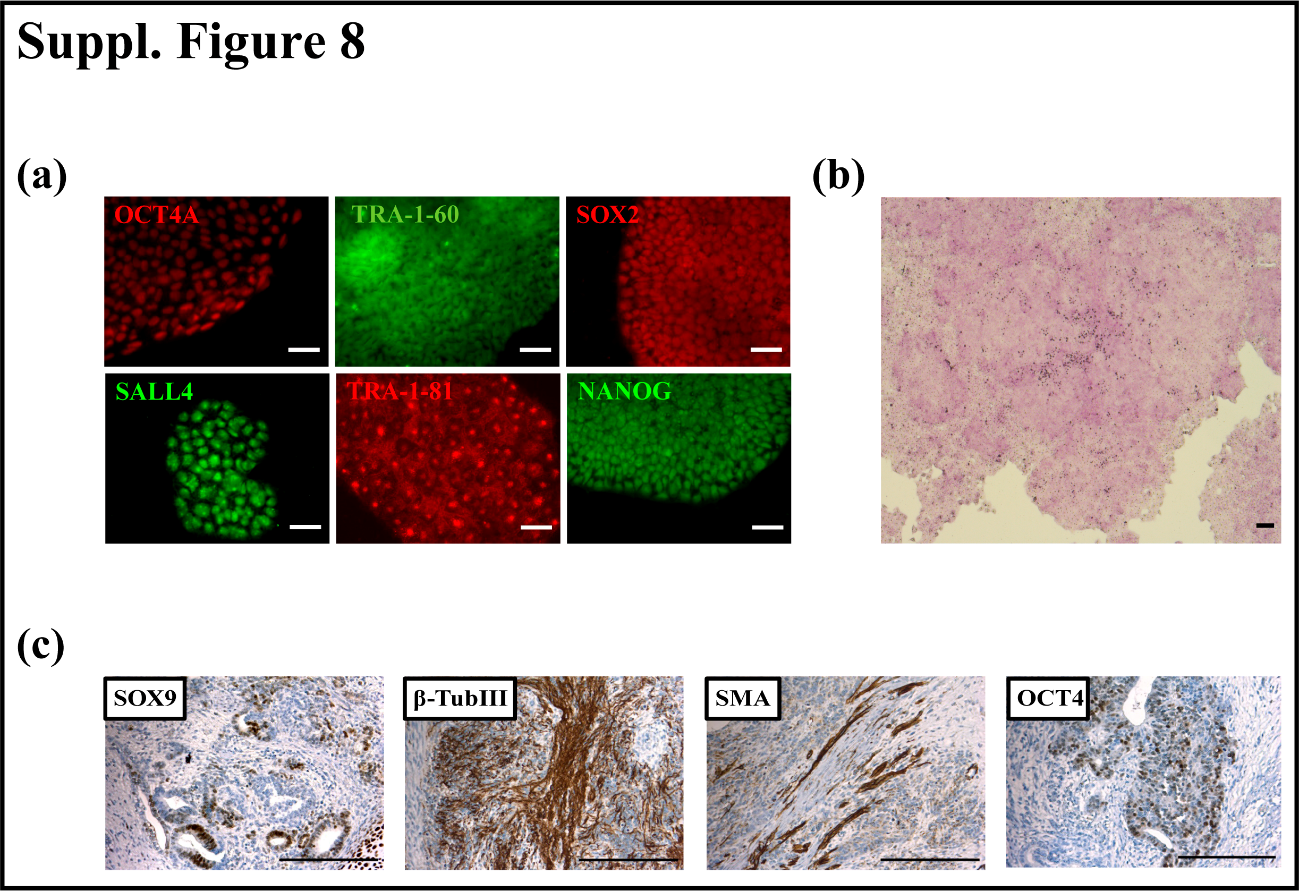


**Suppl. Figure 8:** Characterization of DPZ_iRhpb#4Δpb. **(a)** Immunofluorescence staining. Detection of OCT4A, LIN28, TRA-1-60, SOX2, TRA-1-81 and, SALL4. In DPZ_iRhpb#4Δpb the *piggyBac* reprogramming construct has been removed and, consequently, the expression of all pluripotency factors is of endogenous origin (Scale bar 20 µm). **(b)** Alkaline phosphatase staining of DPZ_iRhpb#4Δpb (Scale bar 100µm). **(c)** Immunohistochemical analysis of DPZ_iRhpb#4Δpb teratoma. Teratoma sections were stained for representative markers of the three germ layers SOX9, β-tubulin III, smooth muscle actin (SMA), and OCT4A to show that the cells remained undifferentiated in the tumor (Scale bar 100 µm).


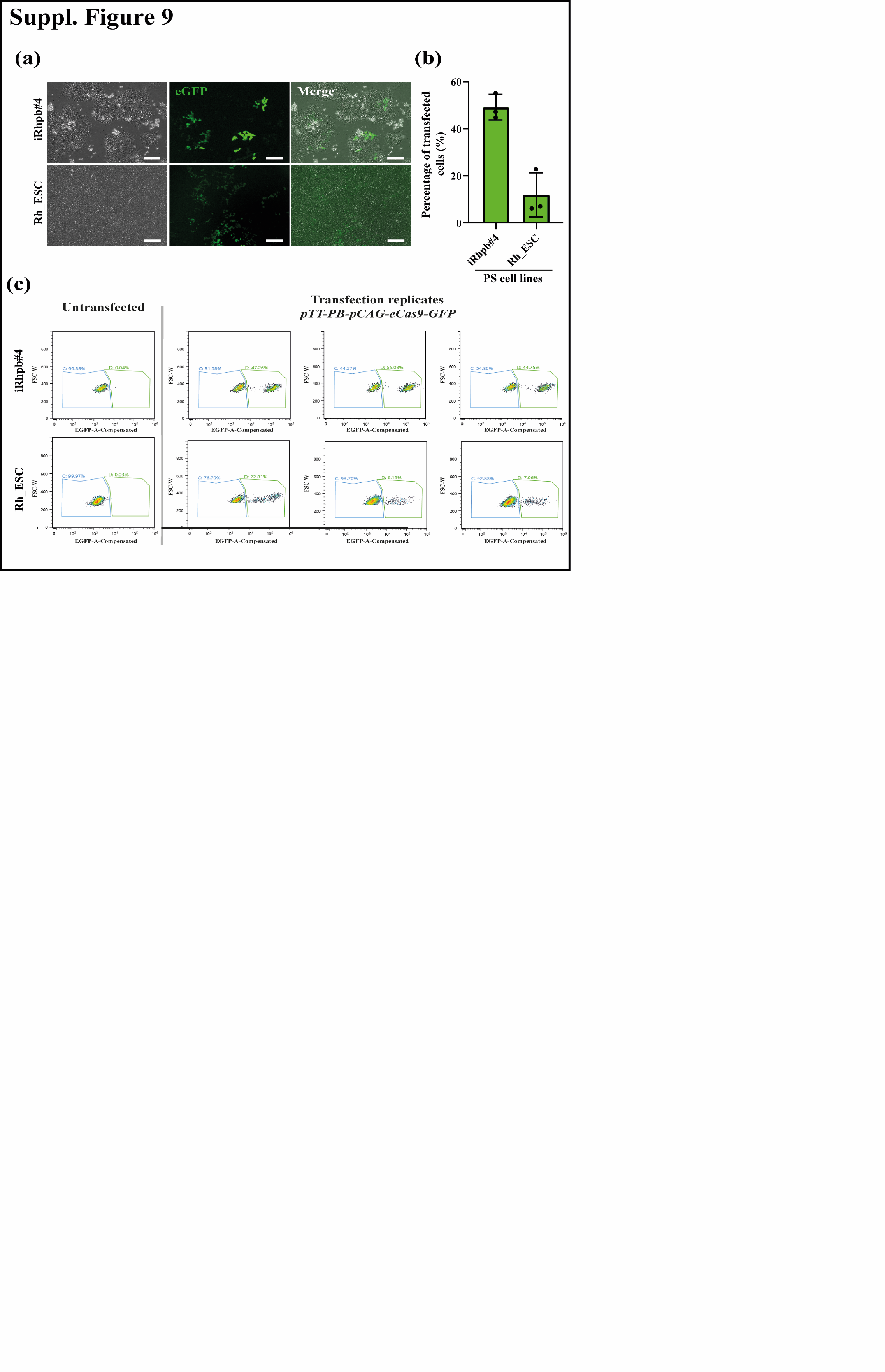


**Suppl. Figure 9:** Transfection efficiency of the *pTT-PB-pCAG-eCas9-GFP-U6-gRNA-Neo* together with *pBase-tdTomato* in rhesus iPSC. Efficiency was evaluated by fluorescence-activated cell sorting (FACS). **(a)** Representative images (bright-field, fluorescence and merge) of a transfection for each cell line (iRhpb#4 and Rh_ESC) (day 5 after transfection) (Scale bars 100µm). **(b) (c)** Three transfections per cell line were analyzed to calculate the percentage of transfected cells. FACS analysis was performed according to the expression of the fluorescent reporter in the transposase vector (eGFP+ cells). **(b)** Graph representing the efficiency (Mean ±SD) (n=3+3). **(c)** Gating strategy used for sorting including as negative control untransfected cells (x-axis eGFP Compensated) (y-axis. Forward scatter-width).


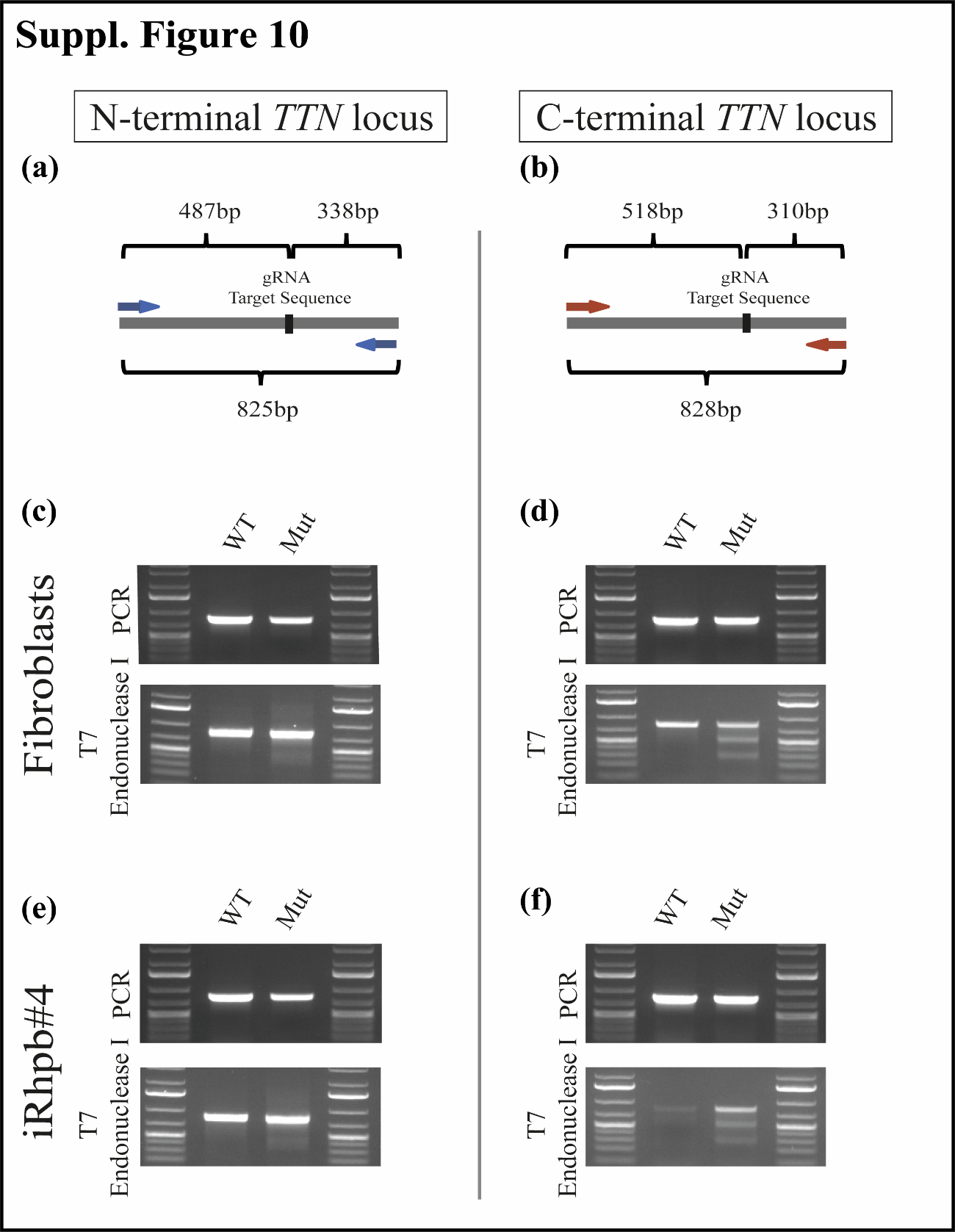


**Suppl. Figure 10:** Bulk population analysis by T7 endonuclease I assay of *TTN* N- and C-terminal target *loci*. One iPSC line (iRhpb#4) and primary macaque fibroblasts were transfected with the respective editing vectors (Mut). Non-transfected cells were used as negative control (Wildtype, WT). **(a) (b)** Graphical representation of the PCR product containing the potentially mutated loci. **(c) (d) (e) (f)** Upper gel image shows PCR products. PCR amplicons were purified, denatured and rehybridized before T7 endonuclease digestion (lower gel). The presence of bands with a smaller size than the original PCR amplicon evidences the presence of INDEL mutations in the CRISPR-Cas treated population (marker 1kb Plus DNA ladder, NEB).


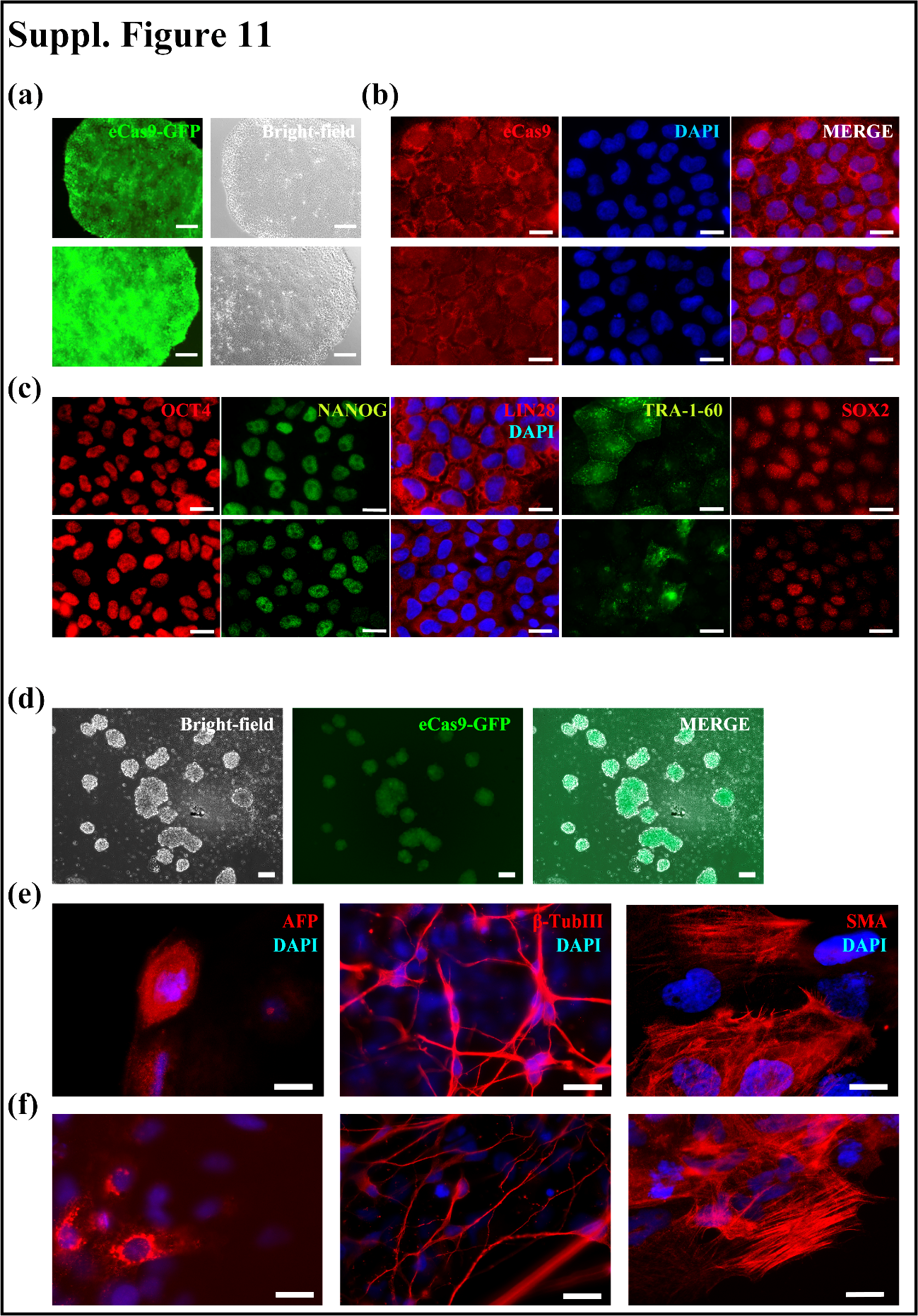


**Suppl. Figure 11:** Isogenic controls generated by the transfection of DPZ_iRhpb#4 (upper panel) and Rh_ESC (lower panel) with pCAG-eCas9-GFP-U6-gRNA-Neo. The lines constitutively express eCas9 and GFP but no gRNA. **(a)** Fluorescence microscopy of cell lines and **(b)** staining for eCas9 (Scale bars, (a)-100 µm and (b)-20 µm). **(c)** Immunofluorescence staining for the detection of pluripotency markers OCT4A, NANOG, LIN28, TRA-1-60 and SOX2 (Scale bar 20 µm). **(d) (e) (f)** Embryoid body (EB) formation of the two isogenic control lines generated. **(d)** Floating EB express eCas9-GFP at day 4 of differentiation, checked by fluorescent microscopy, exemplarily shown for DPZ_iRhpb#4::eCas9-GFP (Scale bar 100 µm). **(e) (f)** Outgrown EBs were stained for representative markers of the three germ layers, α-fetoprotein (AFP), β-Tubulin III, and α-smooth muscle actin (SMA) (Scale bar 20 µm).


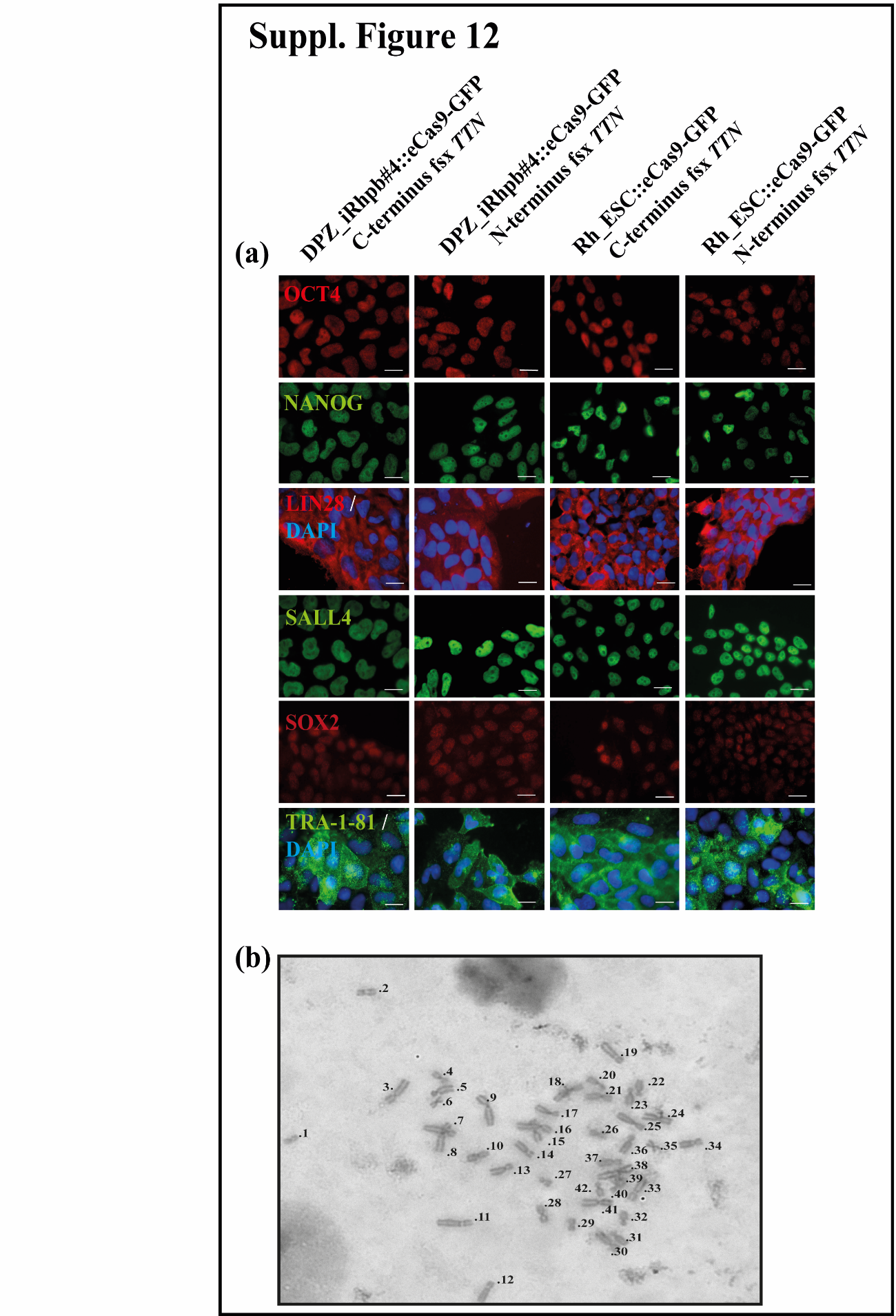


**Suppl. Figure 12:** **(a)** Immunofluorescence staining of DPZ_iRhpb#4 and Rh_ESC containing single truncation mutations in the N- and C-terminal parts of the *TTN* gene (rhesus exons homologous to human *TTN* exons 38 (N-terminus) and 280 (C-terminus); ENST00000589042.5). Detection of OCT4A, NANOG, LIN28/DAPI, SALL4, SOX2 and TRA-1-81/DAPI (Scale bar 20 µm). **(b)** All cell lines show normal chromosomal counts. DPZ_iRhpb#4::eCas9-GFP C-terminus *fsx TTN* is exemplarily shown (passage 64).


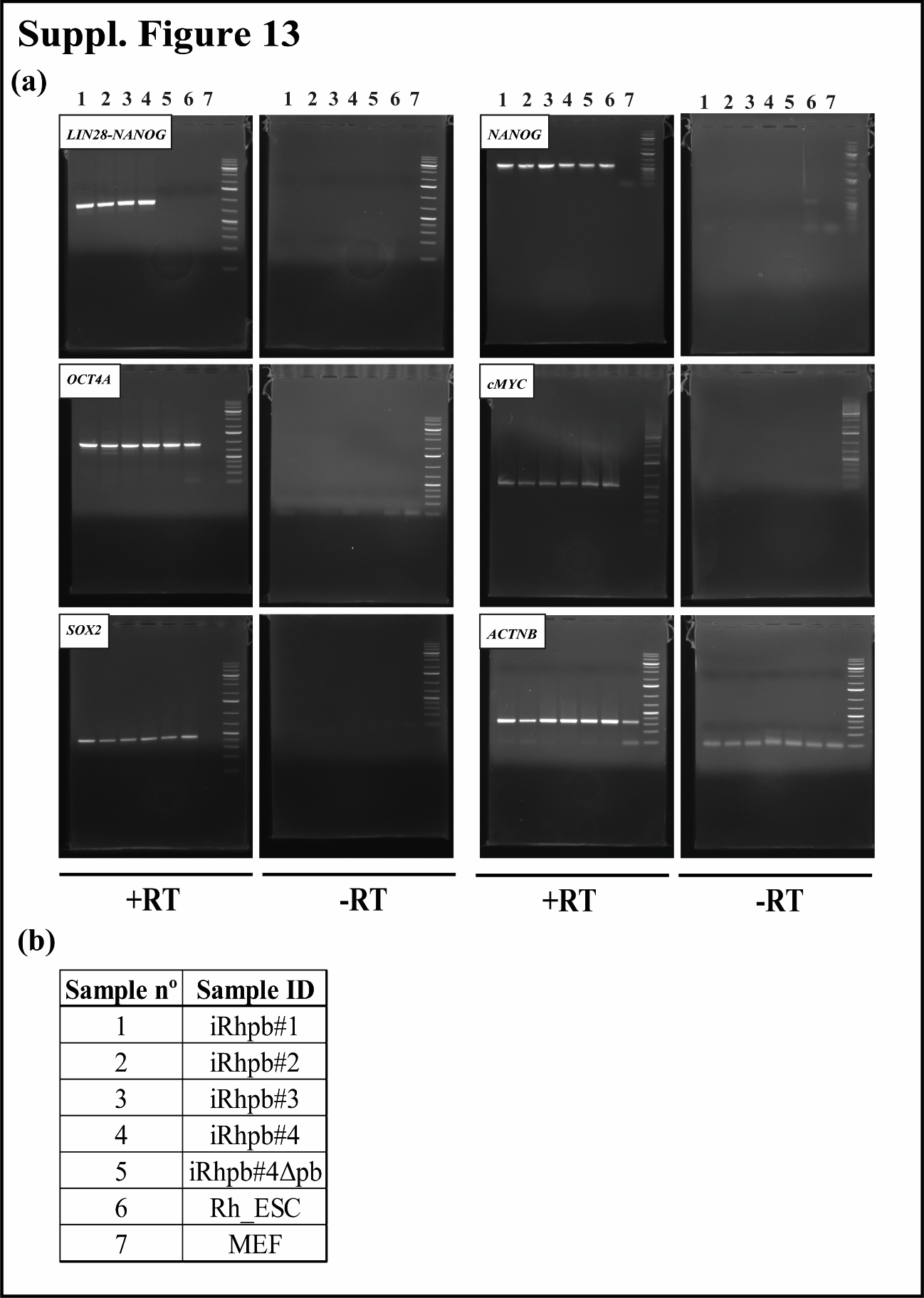


**Suppl. Figure 13:** **(a)** Original gel pictures for the RT-PCR performed to evaluated pluripotency factor expression on the transcript level (Fig. 4b). Amplicon size and PCR conditions detailed in (Fig. 4 and Suppl. Table 1) **(b)** Sample identification in the different gels.


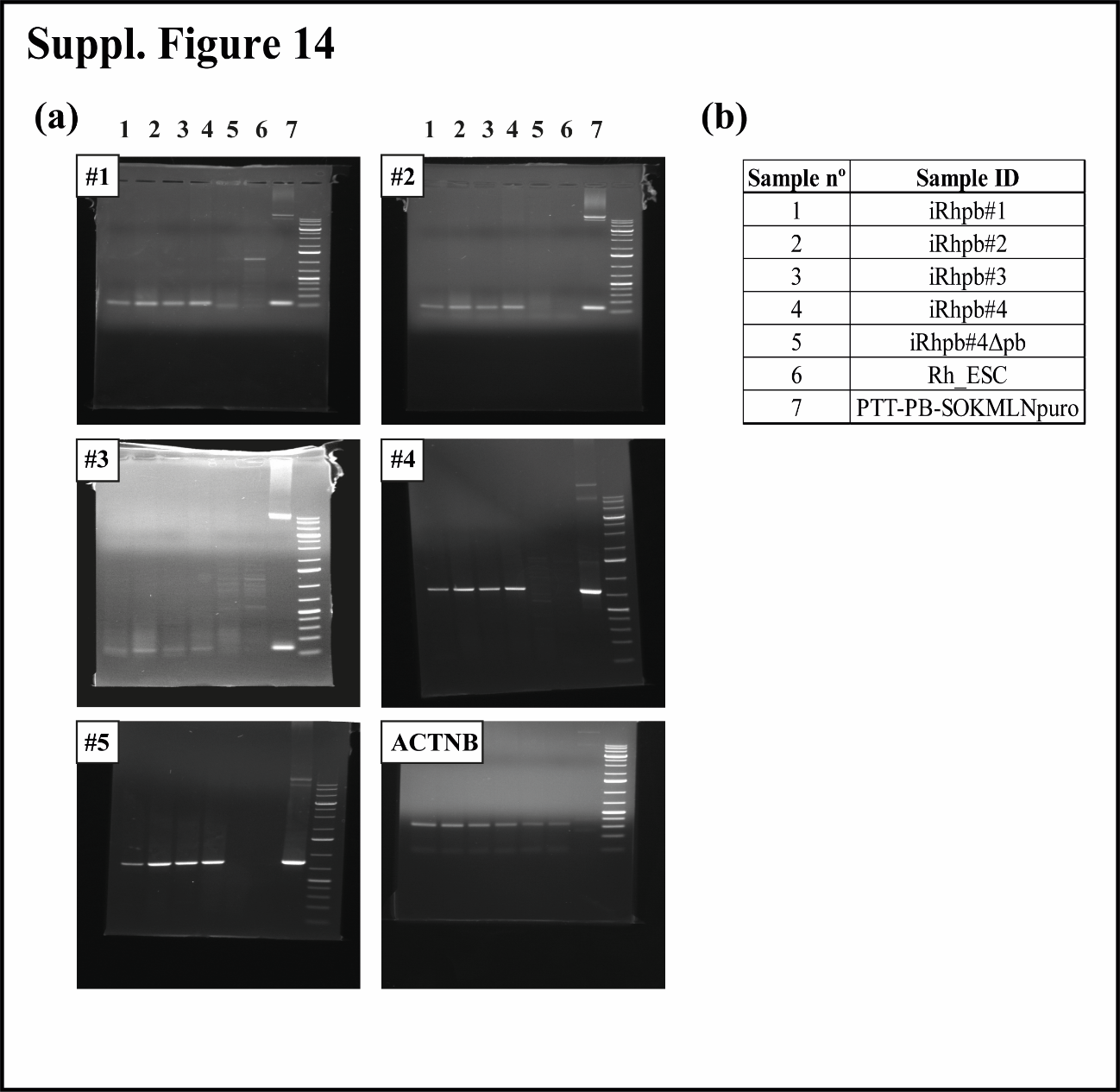


**Suppl. Figure 14:** **(a)** Original gel pictures for the PCR performed in order to detect the presence of the exogenous-factor coding piggyBac transposon (Suppl. Figure 3). Amplicon size and PCR conditions detailed in (Suppl. Figure 3 and Suppl. Table 1) **(b)** Sample identification in the different gels.


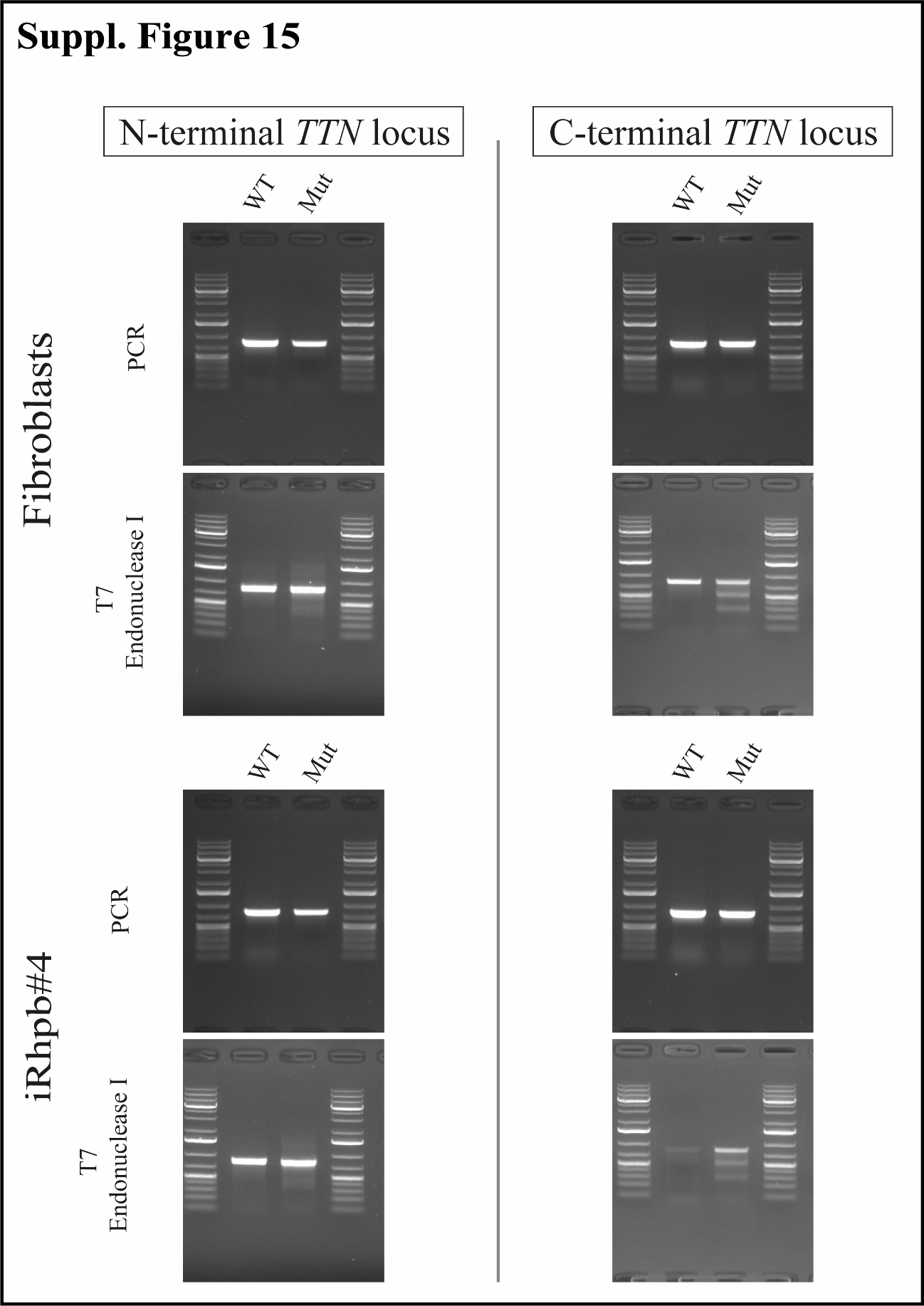


**Suppl. Figure 15:** **(a)** Original gel pictures for T7 endonuclease assay. The picture includes PCR gels (amplicon size and PCR conditions detailed in Suppl. Figure 10 and Suppl. Table 1) and gels after digestion of the PCR product with T7 endonuclease I (expected band size detailed in Suppl. Figure 10)
